# Resin bees of genus *Megachile*, subgenera *Callomegachile* and *Carinula* (Hymenoptera, Megachilidae) from Thailand with description of a new species

**DOI:** 10.3897/zookeys.997.34935

**Published:** 2020-11-25

**Authors:** Nontawat Chatthanabun, John S. Ascher, Nantasak Pinkaew, Chawatat Thanoosing, Prapun Traiyasut, Natapot Warrit

**Affiliations:** 1 Center of Excellence in Entomology and Department of Biology, Faculty of Science, Chulalongkorn University, Bangkok 10330, Thailand Chulalongkorn University Bangkok Thailand; 2 Insect Diversity Lab, Department of Biological Sciences, National University of Singapore, 16 Science Drive 4 S3 Level 4, 117558, Singapore National University of Singapore Singapore Singapore; 3 Department of Entomology, Faculty of Agriculture at Kamphaengsaen, Kasetsart University, Kamphaengsaen Campus, Nakhon Pathom, 73140, Thailand Kasetsart University Bangkok Thailand; 4 Department of Life Sciences, The Natural History Museum, Cromwell Road, London SW7 5BD, UK The Natural History Museum London United Kingdom; 5 Department of Life Sciences, Imperial College London, South Kensington Campus, Exhibition Road, London SW7 2AZ, UK Imperial College London London United Kingdom; 6 Program in Biology, Faculty of Science, Ubon Ratchathani Rajabhat University, Ubon Ratchathani, 34000, Thailand Ubon Ratchathani Rajabhat University Ubon Ratchathani Thailand

**Keywords:** Apoidea, Megachilini, Pollinator, Southeast Asia

## Abstract

Resin bees of the genus Megachile subgenus Callomegachile sensu lato (Hymenoptera; Megachilidae) from Thailand are reviewed. The 14 species treated include those described or revised in the subgenus Alocanthedon, a junior synonym of *Callomegachile* (three species), and in *Carinula* (one species). One new species is described, *Megachile chiangmaiensis* Chatthanabun and Warrit, **sp. nov.** The replacement name *Megachile parornata* Chatthanabun, Warrit and Ascher, **nom. nov.**, is proposed for *M. gigas* Wu (not Schrottky), which is recorded for the first time outside China. For each species, maps and full label data for the examined material documenting occurrences in Thailand are provided. In addition, global ranges, floral associations, and other life history data are summarized and a key to the Thai species is provided for females.

## Introduction

Bees in the subgenus Callomegachile Michener, 1962, of the genus *Megachile* Latreille, 1802 (both sensu lato of Michener 2007) are resin and mud-collecting megachilines native to the Old World that vary from moderate in size to gigantic for a bee (9.0–40.0 mm) and have an elongate, parallel-sided “chalicodomiform” body shape. *Callomegachile* are widespread in the Old World where native to the Paleotropics including all of sub-Saharan Africa, most of Asia extending northwards to subtropical and temperate latitudes, and the Australian region. At least 115 described species of *Callomegachile* sensu lato are currently known (Ascher and Pickering 2020; including 8 species of subgenus Carinula Michener, McGinley & Danforth, 1994) making it the most species-rich subgenera of resin bees and the second most species-rich overall (after the leafcutter bees subgenus Eutricharaea Thomson, 1872) in the genus *Megachile* (the third most species rich bee genus with 1490 valid species globally; Ascher and Pickering 2020), Most *Callomegachile* species collect plant resins as material for nest construction, hence the common name resin bees. In common with other species of Michener’s (2007)*Megachile* Group 2, species of *Callomegachile* do not fold regularly-cut leaves to make their nests (a synapomorphy of his Group 1, corresponding to genus *Megachile* sensu stricto (Litman et al. 2011; Trunz et al. 2016; Gonzalez et al. 2019)), but some can incorporate irregularly-cut leaf pieces into nests, especially for making closures (Michener 2007).

In the New World, Megachile (Callomegachile) umbripennis Smith, 1853 is adventive on several Pacific Islands including the Hawaiian Islands and also locally in the Western Hemisphere (Michener 2007; Gonzalez and Engel 2012) where recently detected in South Florida (Ascher et al. 2016). Megachile (Callomegachile) sculpturalis Smith, 1853 of East Asia is now adventive in Eastern North America and widely distributed, having been found in southern Canada and in all of the Eastern United States (and in the Central States as far west as Nebraska, Kansas, and Texas). In addition, Megachile (Callomegachile) rufipennis (Fabricius, 1793) and M. (Carinula) torrida Smith, 1853 are adventive in the West Indies.

As with the delimitation of *Megachile* sensu lato, there has been considerable variation in subgeneric classification of *Callomegachile*, with Michener (2000, 2007) proposing a very inclusive concept of the group while noting the distinctiveness of several named lineages. However, other authors have partitioned these bees more narrowly, with Engel and Gonzalez (2011) describing a new subgenus Alocanthedon Engel & Gonzalez, 2011 based in part on clypeal shape in females and the presence of dense patch of black setae on male forewing. However, a recent molecular phylogenetic analysis (Trunz et al. 2016) treated *Alocanthedon* as a junior synonym of *Callomegachile*, which we follow here, pending more thorough investigation of relationships between the African and Asian taxa and, in particular, of the status of the gigantic *Megachile* such as *M. pluto* Smith, 1860 for which the name *Eumegachilana* Michener, 1965 is available (see Michener 2007).

In an analysis that resolved the traditional subgenus Callomegachile sensu lato (sensu Michener 2007) as polyphyletic, Trunz et al. (2016) found that *Carinula*, including the well-known Asian species *M. stulta* Bingham, 1897, were well separated from typical *Callomegachile*. For sake of completeness, we treat in this work all Thai resin bees including both M. (Carinula) and M. (Callomegachile) in the narrower sense, but with the former recognized as a separate subgenus in light of its divergent placement in Trunz et al.’s (2016) phylogenetic trees. It has distinctive characters such as a complete longitudinal median clypeal carina in females and lack of a front coxal spine in males (Michener 2007).

Morphological characters uniting *Callomegachile* sensu Michener (2007) include striate arrangement of punctures on mesoscutum and lower part of mesepisternum; mandibles of female bees usually equipped with 3 to 7 teeth with ridges that are minutely roughened (less so in *Carinula*, as noted by Michener 2007); small appressed hairs present on inner mandibular surface of adductor interspace. In males, the sixth metasomal tergum (T6) is weakly bilobed or lacks median emargination, and the gonoforcep is slender (broadened in *Carinula*) (Michener 1962, 1965, 2007).

Although many *Callomegachile* species are relatively well known and easy to recognize as such, more taxonomic and phylogenetic work is needed to clarify both subgeneric and species limits and to document newly discovered species. Many species described historically have poor original descriptions, are highly variable morphologically (especially in hair color), and have been inaccurately or controversially classified. In addition, the *Callomegachile* sensu lato fauna of Southeast Asia, including Thailand, remains poorly documented, in spite of their abundance and general distribution across the region (Gonzalez and Engel 2012; Ascher et al. 2016). Furthermore, interpretation of their biogeography has been complicated by their propensity to become adventive both across oceans and, potentially, within Asia itself (see Ascher et al. 2016; Soh et al. 2016).

Here, we summarize the occurrence of *Callomegachile* sensu lato species in Thailand from (1) literature records (2) historical specimens in two of the largest insect collection facilities in Thailand (3) the National University of Singapore, Division of Biological Sciences, Insect Diversity Lab database and (4) specimens recently collected from numerous collecting trips from 2006–2019. Distributions records, maps of Thai distributions, floral records, measurements, and images of pinned vouchers are provided based on study of both historical and recently collected specimens. Image records available for six species on the citizen science portal iNaturalist were also reviewed. One *Callomegachile* species new to science is described along with two new records for Thailand, and a replacement name is proposed for a species described from China and newly detected in Thailand.

## Materials and methods

Within Thailand, 304 *Callomegachile* specimens (169♀, 135♂) were examined, which were deposited at the Natural History Museum of Chulalongkorn University (**NHMCU-BSRU**; 147♀, 130♂), Bangkok, the Department of Entomology and Plant Pathology, Faculty of Agriculture, Chiang Mai University (**CMU**; 5♀), Chiang Mai, the Department of National Parks, Wildlife and Plant Conservation (**DNP**; 4♀), Bangkok, the Kasetsart Kamphaeng Saen Insect Collection (**KKIC**; 13♀, 5♂), Nakhon Pathom, Thailand. Additional specimens were examined in the Insect Diversity Lab Collection of the National University of Singapore including material on loan from the Oberösterreichisches Landesmuseum of Linz, Austria (curator Fritz Gusenleitner, including material assembled by Maximilian Schwarz). Type repositories used for comparison with specimens examined in this study are abbreviated as follows:

**IZB** Chinese Academy of Science, Yunnan, China

**MFNB** Museum für Naturkunde, Berlin, Gemany

**NHMUK** The Natural History Museum, London, United Kingdom

**NMNH** National Museum of Natural History, Smithsonian’s Institution Washington DC, USA

**USA–SEMC** Snow Entomological Museum Collection, Lawrence

**ZMUC** Zoological Museum of the University of Copenhagen, Copenhagen, Denmark

Types of the valid species-group taxa were examined to the extent possible, including those of some but not at all of the names in synonymy. Image records on the citizen science portal iNaturalist were reviewed and identified by JSA.

Specimens were examined and measured with a Zeiss Stemi 508 dissecting microscope equipped with an ocular micrometer or calipers. Body length was measured from edge of clypeus (in dorsal view) to apex of T6. Forewing length was measured from tegula to lateral wing margin. Interocellar distance (ID) and ocelloccipital distance (OD) were measured and these distances were calculated into ID/OD proportion. Male genitalia were dissected following a method modified from Gonzalez (2008). Photomicrographs were prepared using a Canon 7D Mark II digital camera attached to a T2-T2 1.6× SLR long-distance microscope lens, and were processed with Adobe Photoshop CS6. Measurements are reported in millimeters using Axiovision LE 4.8.2.0. Bees were placed in an insect relaxing jar for 3–4 days to soften the specimens for facilitating the examination of mandibles and labrums. Mandibular teeth are numbered sequentially starting from apex toward the base of the mandible. Morphological terminology follows that of Michener and Fraser (1978), Engel (2001), and Michener (2007).

Specimens from NHMCU-BSRU were collected during 1956–1971 (33♀, 4♂) mostly by Dr. Kloom Vajropala of NHMCU-BSRU and 2006–2019 (112♀, 126♂) from recent surveys by the Thai authors. Label data including localities were recorded verbatim for each specimen and, following georeferencing if necessary, were used to construct distribution maps using Adobe Illustrator. Coordinates for the maps were generated based on specimen labels or, for records lacking GPS data, by georeferencing localities using Google Earth and GPS Geoplaner online. To obtain data on global distribution by country and primary subdivisions, literature records were critically reviewed from Tadauchi and Tasen (2009), Ascher et al. (2016), and the Discover Life Bee Species Guide and World Checklist (Ascher and Pickering 2020), and new records obtained were validated for future inclusion in the latter source. Type localities and repositories are cited, with details provided for those species described from Thailand including new records.

## Taxonomy

### Genus Megachile

Taxon classificationAnimaliaHymenopteraMegachilidae

Latreille

B6CB0435-13D9-5BD5-915B-899A69A45505

Megachile Latreille, 1802: 413, 433. Type species: Apis centuncularis Linnaeus, 1758, by designation of Curtis, 1828, pl. 218.

### Subgenus Callomegachile

Taxon classificationAnimaliaHymenopteraMegachilidae

Michener

0D29FE1E-BF62-5583-B044-DF687D2942A7

Chalicodoma (Callomegachile) Michener, 1962: 21. Type species: Chalicodoma mystaceana Michener, 1962, by original designation.
Chalicodoma (Eumegachilana) Michener, 1965: 191. Type species: Megachile clotho Smith, 1861, by original designation.
Chalicodoma (Morphella) Pasteels, 1965: 537. Type species: Megachile biseta Vachal, 1903, by original designation.
Chalicodoma (Orientocressoniella) Gupta, 1993: 165. Type species: Megachile relata Smith, 1879, by original designation [but Gupta’s description refers to a bee quite different from the nominal type species, see Baker and Engel 2006].
Chalicodoma (Alocanthedon) Engel & Gonzalez, 2011: 53. Type species: Chalicodoma odontophorum Engel, 2011, by original designation.

#### Diagnosis.

Body elongate. Female mandible three to seven teeth with minutely roughened ridge. Punctures on mesoscutum and lower part of mesepisternum striated. Small appressed hairs on inner mandibular surface of adductor interspace present. In males, carina on T6 bilobed or lacks median emargination; posterior margin of T6 simple and without tooth.

#### Comments.

The subgenus Pseudomegachile Friese, 1898 is superficially similar to *Callomegachile*, in term of size and appearance. Females of *Pseudomegachile* can be recognized by the presence of long erected hairs on inner mandibular surface of adductor interspace, whereas males can be easily recognized by the presence of multidentate apical margin of T6.

### Megachile (Callomegachile) atratiformis

Taxon classificationAnimaliaHymenopteraMegachilidae

Meade-Waldo, 1914

DD1DE8D9-84D4-52E7-83D1-9EBD4C5E1414

Figs 1 , 2

Megachile atratiformis Meade-Waldo, 1914: 456; Female syntype (NHMUK, examined) Mergui (Low Tenasserim), Myanmar.
Chalicodoma (Alocanthedon) atratiforme Engel & Gonzalez, 2011: 68–70.

#### Diagnosis.

Female can be easily recognized by its large body size (18–22 mm); black body covered with black hairs throughout (Fig. 2a); clypeus without median tubercle; mandibles four teeth; apical margin of labrum truncate without tooth (Fig. 2d); mesoscutum with weak transverse wrinkle pattern on disc, posteriorly also with weakly transverse wrinkle pattern (Fig. 2e); yellow wings; black scopa. Male can be easily recognized by the presence of black setae patch on medial cell of forewings; apical of T6 with shallow concavity; front tibia modified (Engel and Gonzalez 2011).

**Figure 1.**
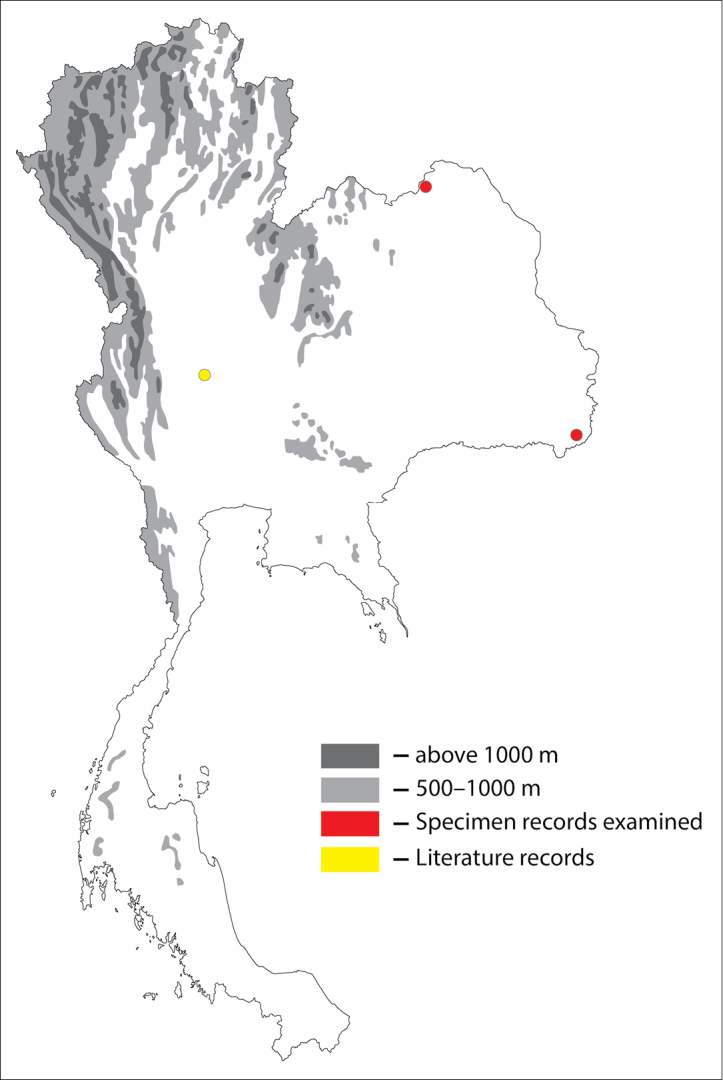
Thai distribution of Megachile (Callomegachile) atratiformis.

**Figure 2.**
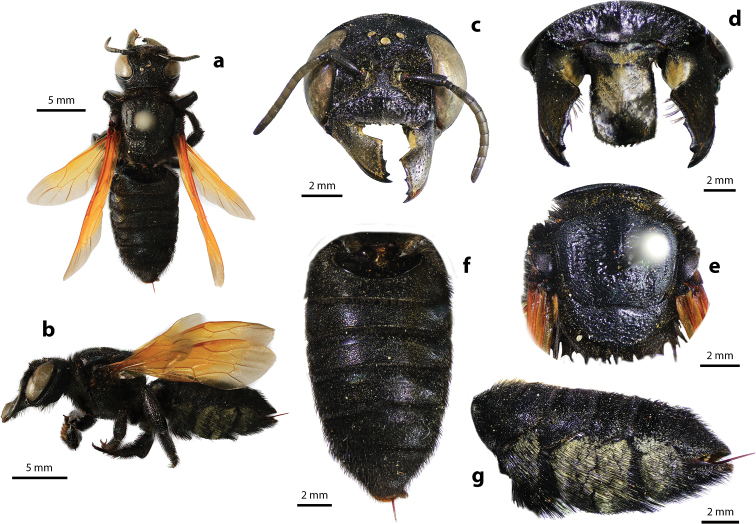
Megachile (Callomegachile) atratiformis Meade-Waldo, 1914, female **a** dorsal view **b** lateral view **c** frontal view **d** frontal view of mandible and labrum **e** dorsal view of mesoscutum **f** dorsal view of metasoma **g** lateral view of metasoma and scopa.

#### Literature records.

Malaysia. Negeri Sembilan, Pahang (Meade-Waldo 1914; Gonzalez and Engel 2011; Ascher and Pickering 2020); Myanmar. Mergui Archipelago in Tanintharyi Region (Meade-Waldo 1914; Gonzalez and Engel 2011; Ascher and Pickering 2020); Thailand. Uthai Thani (Engel and Gonzalez 2011).

#### Material examined.

Female syntype. Myanmar: Tannintharyi Region. “Type H. T.; B.M. TYPE HYM. 2037. 17. a.; *Megachile atrata* (Var), Smith, female; Lower Tenasserim. Mergui, 11–89. C. T. Bingham.; Col. C. T. Bingham 96–30; Megachile (Eumegachile) atratiformis M.W., G. Meade-Waldo det., Type, female; NHMUK 013379843”; Thailand. Nong Khai Province: 1♀, Phon Phisai district, Cowboy Coffee, 18°6'9.95"N, 103°6'17.96"E, Alt. 147.65 m, 15-I-2017, coll. N. Warrit et al. (leg. NC and NW). Ubon Ratchathani Province: 1♀, Phu Jong Na Yoi Nat. P., Kaeng Ka Lao, 14°26'10.98"N, 105°16'37.05"E, Alt. 322 m, 5-I-2019, coll. N. Warrit et al (leg. NC and NW).

#### Floral records.

Engel and Gonzalez (2011) noted the species was captured on an indigenous tree, *Dipterocarpus obtusifolius* Teijsman & Miquel.

#### Comments.

Meade-Waldo’s original material was composite (Engel and Gonzalez 2011) so we exclude from the distribution records from Middle Tenasserim: Haundraw [also known as Houngdarau] Valley in Myanmar (= *M. odontophorum*) and also from Penang in Malaysia.

### Megachile (Callomegachile) disjuncta

Taxon classificationAnimaliaHymenopteraMegachilidae

(Fabricius, 1781)

0559D61D-2DD8-5C44-B637-8A8BFDD05DFF

Figs 3 , 4 , 5

Apis disjuncta Fabricius, 1781: 481. Female type (ZMUC, not examined) “America” [erroneous; surely from Asia, and perhaps from India].
Anthophora disjuncta : Fabricius, 1804: 374.
Trachusa disjuncta : Jurine, 1807: 251.
Megachile disjuncta : Lepeletier, 1841: 331.
Megachile (Pseudomegachile) disjuncta : Friese, 1911: 207.
Chalicodoma (Callomegachile) disjuncta : Michener, 1965: 191.

#### Diagnosis.

Female can be recognized by its medium body size (13–18 mm); black body covered with black hairs throughout, except propodeal triangle and T1 with white hairs (Fig. 4a); apical margin of clypeus with two small tubercles (Fig. 4d); mandibles five teeth with two stout apical teeth at apex and three small teeth basally; labrum rectangle; apical margin of clypeus truncate with two lateral teeth (Fig. 4e); wings hyaline except fuscous apical part; black scopa (orange in part in females of the superficially similar leafcutter bee M. (Aethomegachile) conjuncta Smith, 1853 which also has a shorter and less parallel-sided metasoma, finer tergal punctures, and cutting edges on mandibles). Male is similar to female except paraocular area and apical margin of clypeus with white hairs (Fig. 5c); mandibles three teeth; labrum rectangle with round corners (Fig. 5d).

**Figure 3.**
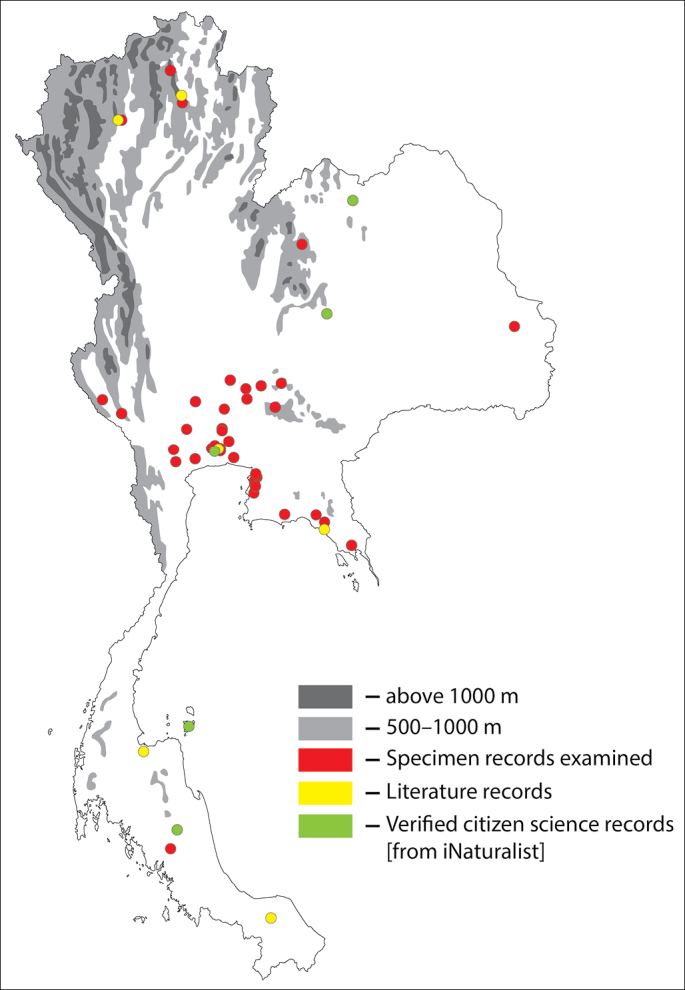
Thai distribution of Megachile (Callomegachile) disjuncta.

**Figure 4.**
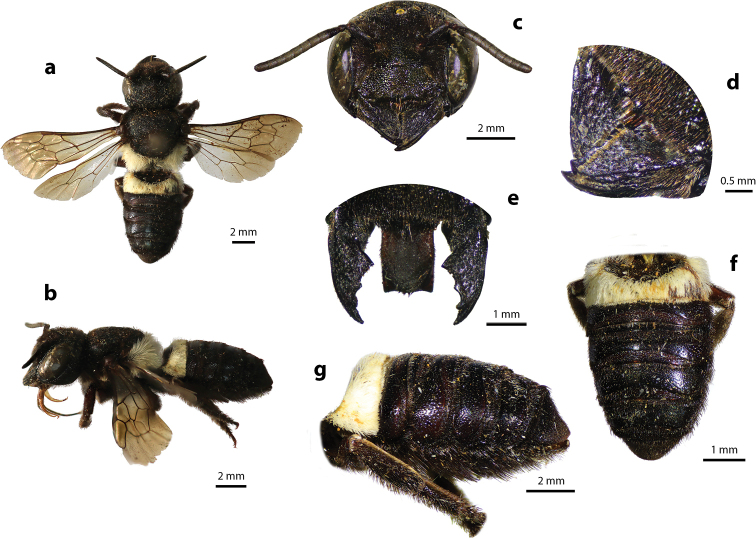
Megachile (Callomegachile) disjuncta (Fabricius, 1781), female **a** dorsal view **b** lateral view **c** frontal view **d** oblique view of clypeal margin **e** frontal view of mandible and labrum **f** dorsal view of metasoma **g** lateral view of metasoma and scopa.

#### Literature records.

China. Anhui, Beijing, Fujian, Guangxi Zhuang, Guizhou, Hainan, Hebei, Hunan, Jiangsu, Jiangxi, Shandong, Shanghai, Shanxi, Sichuan, Zhejiang; India. Andaman and Nicobar Islands: Long Island (notable records from Cockerell 1912), Andhra Pradesh, Chandigarh, Haryana, Karnataka, Maharashtra, Puducherry, Tamil Nadu, Uttarakhand (Lepeletier 1841; Smith 1873; Gribodo 1884; Bingham 1897; Cameron 1907; Friese 1911; Cockerell 1912; Cockerell 1919; Flectcher 1920; Dover 1921; Schulthess-Rechberg 1935; Ascher et al. 2016); Indonesia. Java, Sumatra (Gribodo 1884; Schulthess-Rechberg 1935; Ascher et al. 2016); Madagascar. Antananarivo, Bourbon, Toamasina (Gribodo 1884; Grandidier 1890; Pauly 2001; Ascher et al. 2016); Malaysia. Kelantan, Kepong, Kuala Lumpur, Negeri Sembilan, Penang, Selangor, (Ascher et al. 2016); Mauritius. Rodrigues Island (Smith 1873; Gribodo 1884; Bingham 1897; Cameron 1907; Pauly 2001; Ascher et al. 2016); Myanmar. Tenasserim (Bingham 1897; Flectcher 1920; Dover 1921; Schulthess-Rechberg 1935); Reunion. (Pauly 2001; Ascher et al. 2016); Seychelles. Mahe, Morne Blanc, Praslin Island (Cameron 1907; Cockerell 1912; Pauly 2001; Ascher et al. 2016); Singapore. (Ascher et al. 2016); Sri Lanka. Hambantota, Puttalam (Ascher et al. 2016); Vietnam. Thai Binh (Ascher and Pickering 2020). Additional records based on images on iNaturalist are from Bangkok, Chaiyaphum, Surat Thani, and Udon Thani Provinces (credit: lennyworthington 2017; nopcoeur 2018; bernhard_hiller 2019; tonykris 2019; alexeyreshchikov 2020).

**Figure 5.**
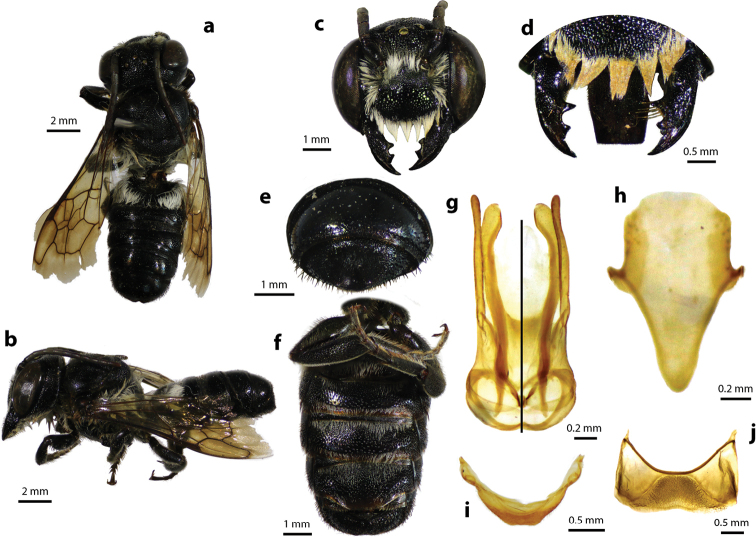
Megachile (Callomegachile) disjuncta (Fabricius, 1781), male **a** dorsal view **b** lateral view **c** frontal view **d** frontal view of mandible and labrum **e** frontal view of T7 **f** ventral view of metasomal sterna **g** dorsal (left) and ventral (right) views of penis **h** dorsal view of S8 **i** T7 **j** S5.

#### Material examined.

Thailand. Ayutthaya Province: 1♀, XI-1961, coll. unknown (leg. NC and NW); 1♀, Beung Pra ram, 01-VIII-1970, coll. unknown (leg. NC and NW). Bangkok Province: 1♀, Phra Nakhon, 05-I-1960, coll. unknown (leg. NC and NW); 1♀, Phra Nakhon, 12-VII-1960, coll. unknown (leg. NC and NW); 1♀, 15-II-1963, coll. unknown (leg. NC and NW); 1♀, Nong Khaem district, 16-IX-1966, coll. Wanida (leg. NC and NW); 1♀, Bang Khen district, 19-VII-1969, coll. unknown (leg. NC and NW); 1♀, Thonburi district, 01-VII-1970, coll. unknown (leg. NC and NW); 1♀, Sa Torn district, Thungmahamek subdistrict, 22-IV-1971, coll. unknown (leg. NC and NW); 1♀, 05-VIII-1971, coll. unknown (leg. NC and NW); 1♀, Sathorn district, Soi Suanglu1, 05-X-2009, coll. K. Attasopa (leg. NC and NW); 1♀, Sathorn district, Soi Suanglu1, 06-X-2009, coll. K. Attasopa (leg. NC and NW). Chanthaburi Province: 1♀, Tha Mai district, 19-IX-1969, coll. unknown (leg. NC and NW); 1♀, Makam district, 25-III-2015, coll. N. Chatthanabun (leg. NC); 1♀, 08-V-1956, coll. unknown (leg. NC and NW); 1♀, Bang Pra district, 08-X-1963, coll. unknown (leg. NC and NW); 1♀, Ang Sila, 04-VII-1970, coll. Nonglak (leg. NC and NW); 1♀, Sriracha district, Bang Pra Reservoir, 04-VII-1971, coll. unknown (leg. NC and NW); 1♀, Mueang district, Soi Na Khao Bor Yang, 13°6'10.2066"N, 100°57'59.5764"E, Alt. 15.86 m, 27-VII-2017, coll. P. Traiyasut (leg. NC and NW). Chiang Mai Province: 1♀, 09-VII-1959, coll. Unknown (leg. NC and NW); 39 ♀, Mueang district, Suthep subdistrict, Faculty of Agriculture Chiang Mai University, 18°47'38.6772"N, 98°57'32.9220"E, Alt. 391 m, 19-VII-2015, coll. Warrit et al. (leg. NC and NW); 17♂, Mueang district, Mae Hia subdistrict, 18°45'51.1272"N, 98°55'39.6192"E, Alt. 232 m, 19-VII-2015, coll. Warrit et al. (leg. NC and NW). Chiang Rai Province: 1♀, Pan district, 17-I-2009, coll. T. Yusing (leg. NC and NW). Loei Province: 1♀, Phu Kradueng district, Phu Kradueng National Park, 16°52'22.4934"N, 101°50'11.7384"E, Alt. 506.28 m, 29-V-2016, coll. Warrit et al. (leg. NC and NW). Kanchanaburi Province: 12♂, Sai Yok district, Wang Krachae subdistrict, 14°9'56.7678"N, 99°3'30.5640"E, Alt. 101.80 m, 24-VI-2016, coll. Warrit et al. (leg. NC and NW); 2♂, Sai Yok district, Wang Krachae subdistrict, 14°11'6.5724"N, 99°3'6.9258"E, Alt. 102.30 m, 24-VI-2016, coll. Warrit et al. (leg. NC and NW). Lop Buri Province: 1♀, 10-IX-2013, coll. Warrit (leg. NC and NW). Nakhon Nayok Province: 1♀, Khao Yai National Park, 06-IX-1970, coll. unknown (leg. NC and NW). Nakhon Pathom Province: 1♂, Kamphaeng Saen district, Kasetsart University Kamphaeng Saen campus, 06-II-2008, coll. Patneti (leg. NC and NW); 1♂, Kamphaeng Saen district, Kasetsart University Kamphaeng Saen campus, 15-VI-2011, coll. Patneti (leg. NC and NW); 1♀, Kamphaeng Saen district, Kasetsart University Kamphaeng Saen campus, 30-XI-2013, coll. Sunita (leg. NC and NW); 1♀, 4♂, Kamphaeng Saen district, 13°44'58.3908"N, 99°52'33.1242"E, Alt. 14 m, 10-VII-2015, coll. Warrit et al. (leg. NC and NW); 1♀, Kamphaeng Saen district, Kasetsart University Kamphaeng Saen campus, 26-I-2016, coll. Noppasiri (leg. NC and NW); 1♀, Kamphaeng Saen district, Kasetsart University Kamphaeng Saen campus, 20-III-2016, coll. K. Laesen (leg. NC and NW); 1♀, Kamphaeng Saen district, Kasetsart University Kamphaeng Saen campus, 29-III-2016, coll. Jirawat (leg. NC and NW); 1♀, Kamphaeng Saen district, Kasetsart University Kamphaeng Saen campus, 14-IV-2016, coll. Adisak (leg. NC and NW); 1♂, Kamphaeng Saen district, Kasetsart University Kamphaeng Saen campus, 11-V-2016, coll. Vilunda (leg. NC and NW); 1♂, Kamphaeng Saen district, Kasetsart University Kamphaeng Saen campus, 04-IX-2016, coll. S. Laengsaruk (leg. NC and NW); 5♀, Kamphaeng Saen district, 14°0'35.1072"N, 100°0'35.9028"E, Alt. 11 m, 12-II-2017, coll. N. Chatthanabun, N. Warrit and V. Sivayyapram (leg. NC and NW). Nakhon Ratchasima Province: 1♀, Pak Chong, 06-I-1964, coll. unknown (leg. NC and NW); 1♀, Pak Chong, 28-VII-1969, coll. W. Sooksri (leg. NC and NW). Nonthaburi Province: 1♀, Pak Kred district, Khlong Phapha, coll. unknown (leg. NC and NW). Pathum Thani Province: 1♂, Ban Ngew district, Samkok subdistrict, Pai rom temple, 18-II-1968, coll. Chairoj (leg. NC and NW); 1♂, 18-II-1968, coll. Rojnee (leg. NC and NW). Phayao Province: 1♂, Mueang district, Mae Ka subdistrict, Phayao University, 03-I-2009, coll. Saowalak (leg. NC and NW); 16♂, Mueang district, Mae Ka subdistrict, Phayao University, 01-VI-2012, coll. Warrit et al. (leg. NW); 1♀, Mae Ka, Phayao University, 19°2'45.3510"N, 99°52'40.5438"E, Alt. 464.26 m, 5-VII-2016, coll. Warrit et al. (leg. NC and NW). Ratchaburi Province: 1♀, 22-VIII-1970, coll. unknown (leg. NC and NW); 1♀, 17-VIII-1973, coll. Pinpong (leg. NC and NW); 2♀, Potharam district, 13°44'50.0070"N, 99°53'38.4180"E, Alt. 6 m, 13-VII-2015, coll. Warrit et al. (leg. NC and NW); 1♀, 01-V-2017, coll. Pornchanok (leg. NC and NW). Rayong Province: 1♀, Ban Pae, 03-VIII-1971, coll. unknown (leg. NC and NW). Samut Prakan Province: 1♂, Park Nam district, 11-VIII-1963, coll. P. Sakulmon (leg. NC and NW). Samut Sakhon Province: 1♂, Banpaew district, 19-VIII-2014, coll. P. Tangtorwongsakul (leg. NC and NW). Saraburi Province: 1♀, 30-VIII-1960, coll. B. Pasook (leg. NC and NW); 1♀, Muak Lek, 03-VIII-1967, coll. unknown (leg. NC and NW); 1♀, Phu Khae, 04-II-1968, coll. Decha (leg. NC and NW); 1♀, Muak Lek, 03-VIII-1971, coll. Unknown (leg. NC and NW). Suphan Buri Province: 1♀, 13-IV-2015, coll. Kaewkan (leg. NC and NW). Trat Province: 1♀, Mueang district, Ao Yai, 23-III-2015, coll. N. Chatthanabun (leg. NC). Trang Province: 1♂, Na Yong district, 7°33'8.0892"N, 99°46'33.6072"E, Alt. 24 m, 11-VI-2015, coll. Warrit et al. (leg. NC and NW). Ubon Ratchathani Province: 1♂, Trakan Phuetphon district, Sarin lake view village, 03-VIII-2014, coll. N. Chatthanabun (leg. NC). Unknown localities: 1♂, 16-VIII-1960, coll. unknown (leg. NC and NW); 1♀, 01-I-1965, coll. unknown (leg. NC and NW); 2♀, 2♂, coll. unknown (leg. NC and NW).

#### Floral records.

Throughout Thailand, M. (Callomegachile) disjuncta can be found abundantly in many agricultural plots that are planted with *Crotalaria juncea* L., a common plant species grown for providing essential nitrogen element to many crop plants in Thailand. Also, a common weed, *Bidens pilosa* L., is frequently visited by the species. One record of M. (Callomegachile) disjuncta was found on *Cratoxylum cochinchinense* (Lour.) Blume.

#### Comments.

Female somewhat superficially resembles M. (Aethomegachile) conjuncta in size and overall appearance.

### Megachile (Callomegachile) faceta

Taxon classificationAnimaliaHymenopteraMegachilidae

Bingham, 1897

B8BF72FE-E1C5-5D23-8511-12D25E864720

Figs 6 , 7 , 8

Megachile faceta Bingham, 1897: 486. Female syntype (NHMUK, examined) Pegu Hills, Myanmar.
Megachile faceta rufojugata : Cockerell, 1931: 2.
Chalicodoma (Callomegachile) faceta : Michener, 1965: 191.

#### Diagnosis.

Female can be recognized by its black medium body size (12–14 mm); vertex and pronotum covered with fulvous hairs; propodeal triangle and T1–T4 with tuft white hairs on lateral edges; apical margin of T5 with white hair band but interrupted at median (Fig. 7a); apical margin of clypeus with two small tubercles (Fig. 7c); mandibles five teeth with two stout apical teeth at apex and three small teeth basally; labrum rectangle with rounded corners (Fig. 7d); vertex with median carina (Fig. 7e); white scopa except black at apical area. Male is similar to female except mandible three teeth; labrum rectangular with shallow impression at median (Fig. 8d).

**Figure 6.**
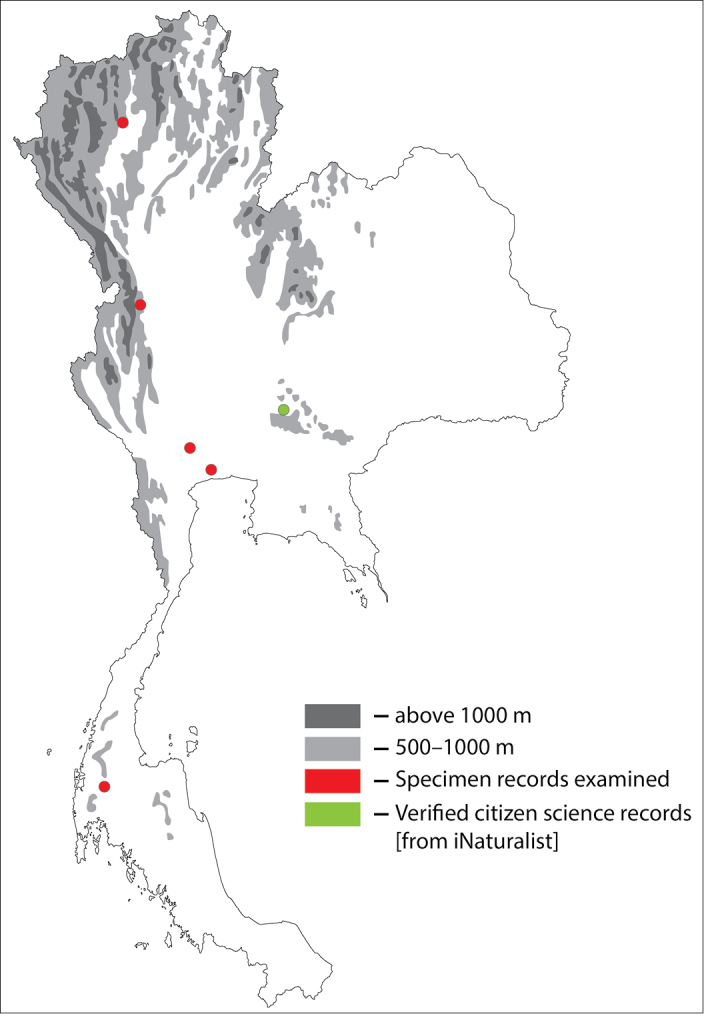
Thai distribution of Megachile (Callomegachile) faceta.

**Figure 7.**
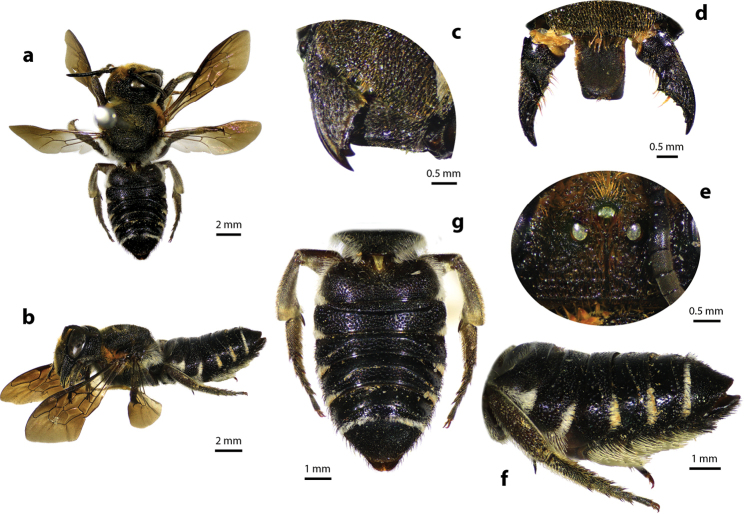
Megachile (Callomegachile) faceta Bingham, 1897, female **a** dorsal view **b** lateral view **c** oblique view of clypeal margin **d** frontal view of mandible and labrum **e** dorsal view of median carina at vertex **f** lateral view of metasoma and scopa **g** dorsal view of metasoma.

#### Literature records.

India. Khasia Hills (Cockerell 1911); Myanmar. Pegu Hills [as Bago Yoma], Tanintharyi Region [as Tenasserim] (Bingham 1897); Taiwan. Sauter (Cockerell 1911). An iNaturalist image shows four females among a group of bees photographed at Hin Tung, Mueang District, Nakhon Nayok Province (credit: scottyastro 2015).

**Figure 8.**
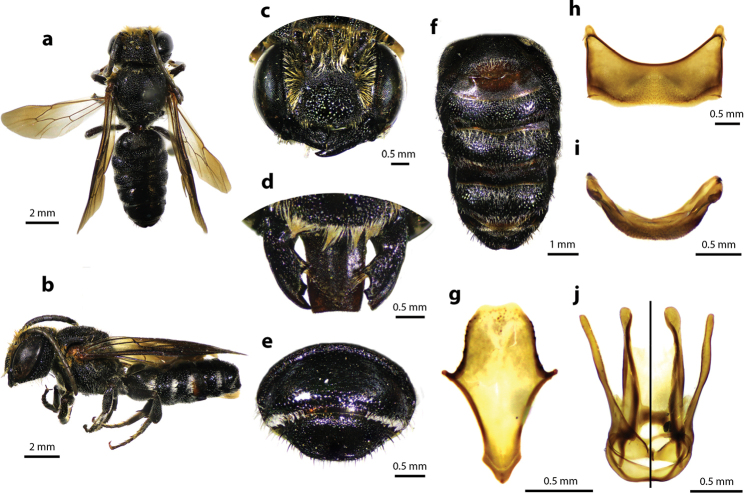
Megachile (Callomegachile) faceta Bingham, 1897, male **a** dorsal view **b** lateral view **c** frontal view **d** dorsal view of mandible and labrum **e** frontal view of T7 **f** ventral view of metasomal sterna **g** dorsal view of S8 **h** S5 **i** T7 **j** dorsal (left) and ventral (right) views of penis.

#### Material examined.

Female syntype. Myanmar. “Type; B.M. TYPE HYM. 2011 17. a.; *Megachile faceta* Bingh. Female, Type; Pegu Hills, Burma, 11-87, Bingham Coll; Col. C. T. Bingham 96–30; NHMUK 013379842”; Thailand. Bangkok Province: 1♀, Pom Prap Sattru Phai district, Khlong Mahanak subdistrict, Sapan Kaw, 18-VII-1971, coll. Sudthida (leg. NC and NW). Chiang Mai Province: 13♂, Mueang district, Mae Hia subdistrict, Mae Hia Agricultural Research, Demonstrative and Training Center, 18°45'51.1272"N, 98°55'39.6192"E, Alt. 232 m, 19-VII-2015, coll. Warrit et al. (leg. NC and NW). Nakhon Pathom Province: 1♀, Kamphaeng Saen district, Kamphaeng Saen subdistrict, Kasetsart University, Kamphaeng Saen Campus, 02-IX-2010, coll. Pakkawat (leg. NC and NW). Nakhon Sawan Province: 2♀, Mae Wong Natural Park, 28-VI-2015, coll. V. Sivayyapram (leg. NC and NW). Surat Thani Province: 1♀, Phanom district, 8°54'35.9460"N, 98°31'37.9590"E, Alt. 118.68 m, 27-I-2018, coll. Warrit et al. (leg. NC and NW).

#### Comments.

The similar Megachile (Callomegachile) facetula Cockerell, 1918, described from Sandakan, Sabah, Borneo, should be looked for in Thailand, but we have not been able to confirm any records. *Megachile strupigera* Cockerell, 1922, from Canton (now Guangzhou in Guandong) in southern China is likely a junior synonym of *M. faceta* based on our examination of images of its type in the NMNH, whereas Wu (2006) placed it in the leafcutter subgenus Amegachile Friese, 1909. Multiple species of leafcutter bees present in the region including Thailand closely resembles *M. facetula* in color pattern, so all identifications must be considered structural characters as well.

### Megachile (Callomegachile) fulvipennis

Taxon classificationAnimaliaHymenopteraMegachilidae

Smith, 1879

2AEA7C55-D85E-5E74-8FA2-8F491C4DDB21

Figs 9 , 10

Megachile fulvipennis Smith, 1879: 68. Female holotype (NHMUK, examined) Nicobar Island, India.
Megachile atratiformis sininsulae Cockerell, 1927: 160.
Chalicodoma (Callomegachile) atratiforme sininsulae : Michener, 1965: 191.
Chalicodoma (Callomegachile) fulvipennis : Michener, 1965: 191.

#### Diagnosis.

Female superficially resembles M. (Callomegachile) atratiformis (Meade-Waldo, 1914), M. (Callomegachile) memecylonae (Engel, 2011) and M. (Callomegachile) odontophora (Engel, 2011), in overall appearance: black body covered with black hairs throughout; yellow wings; black scopa (Fig. 10a, f) except smaller size (14–16 mm); punctures on mesoscutum and lower part of mesepisternum striated; mandibles five teeth with two stout apical teeth at apex and three small teeth basally; labrum rectangular (Fig. 10c).

**Figure 9.**
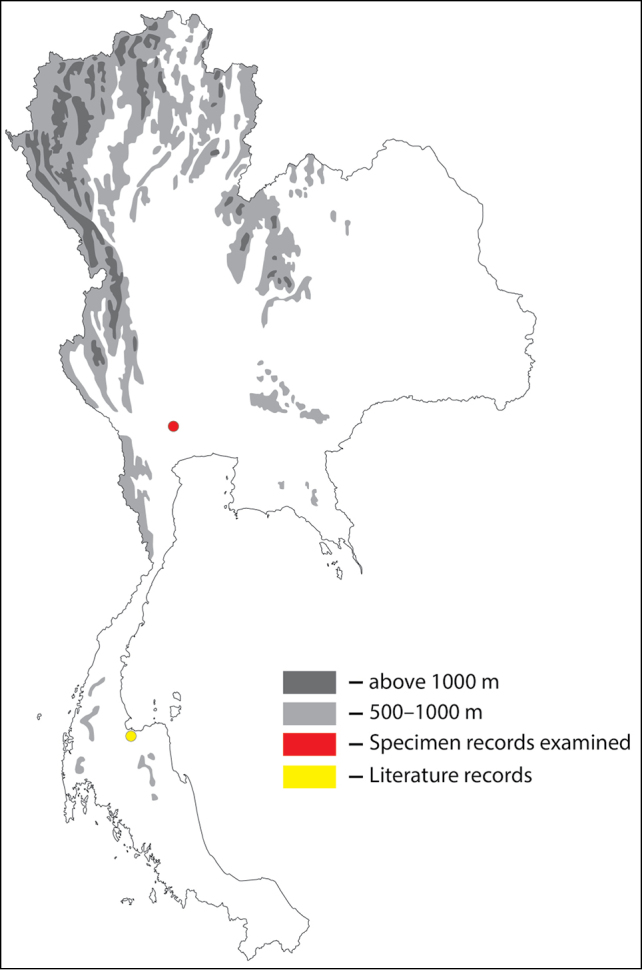
Thai distribution of Megachile (Callomegachile) fulvipennis.

**Figure 10.**
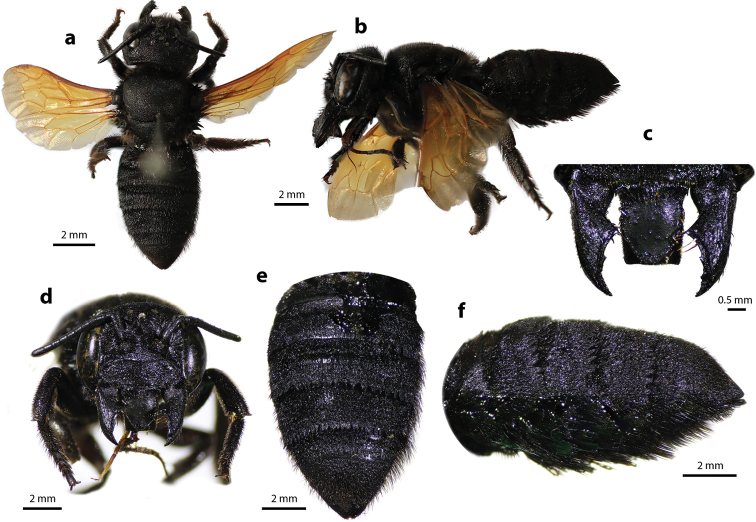
Megachile (Callomegachile) fulvipennis Smith, 1879, female **a** dorsal view **b** lateral view **c** dorsal view of mandible and labrum **d** frontal view **e** dorsal view of metasoma **f** lateral view of metasoma and scopa.

#### Literature records.

India. Andaman and Nicobar Island (Smith 1879; Ascher et al. 2016); Indonesia. Java, Sumatra (Ascher et al. 2016); Malaysia. Perak, Selangor, Terengganu (Ascher and Pickering 2020); Singapore. (Ascher et al. 2016).

#### Material examined.

Female holotype. Nicobar Island [India]. “Holotype; B.M. TYPE HYM. 2055 17. a.; *Megachile fulvipennis* Sm. (Type); Nicobar, 76–30; NHMUK 013379844”; Thailand. Nakhon Pathom Province: 1♀, Kamphaeng Saen district, Kamphaeng Saen subdistrict, Kasetsart University, Kamphaeng Saen Campus, 20-VIII-1956, coll. Chayuta (leg. NC and NW).

### Megachile (Callomegachile) impressa

Taxon classificationAnimaliaHymenopteraMegachilidae

Friese, 1903

D9DA4546-B9E8-5CC9-A6EE-E4941DD75F24

Figs 11 , 12

Megachile impressa Friese, 1903: 358. Male holotype (MFNB, not examined) Tenasserim (Kayin: Thandaung), Myanmar.

#### Diagnosis.

The species superficially resembles M. (Callomegachile) binghami Meade-Waldo, 1912, in terms of its overall appearance and size: black hairs on paraocular area; thorax with white hairs except central area of mesoscutum and scutellum; metasomal terga covered with ferruginous hairs; scopa ferruginous except white basal area; apical margin of clypeus with one medioapical tubercle and two lateral tubercles (Fig. 12b, c); meso- and metatarsi with ferruginous hairs (Fig. 12a). These characters are used to associate male and female bees.

**Figure 11.**
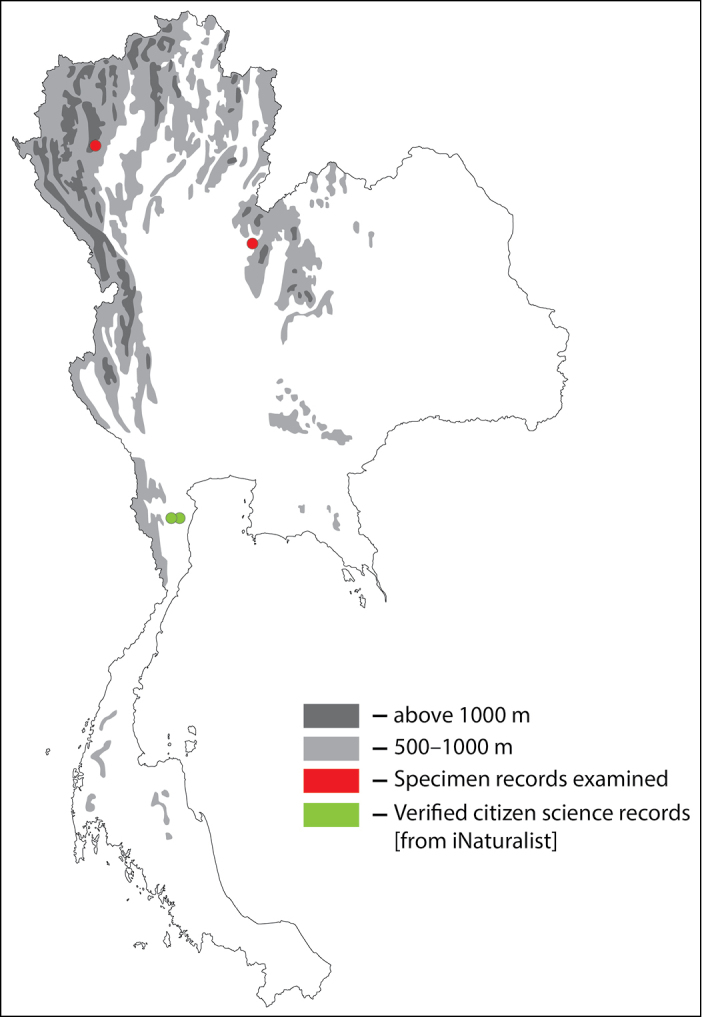
Thai distribution of Megachile (Callomegachile) impressa.

**Figure 12.**
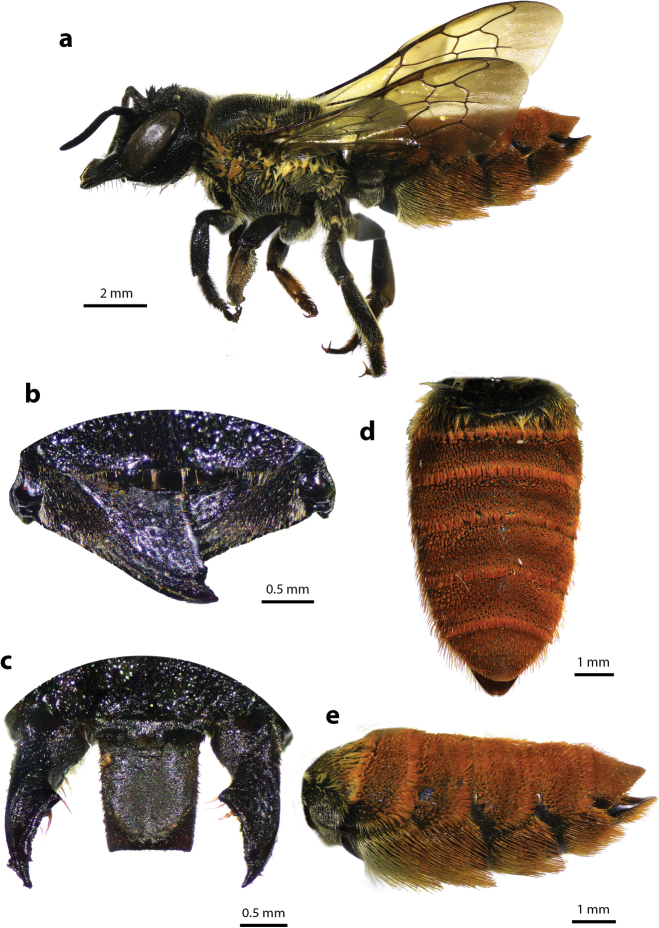
Megachile (Callomegachile) impressa Friese, 1903, female **a** lateral view **b** frontal view **c** clypeus and mandible **d** dorsal view of metasoma **e** lateral view of metasoma.

#### Description.

***Female*.** Length. Total body length 14.28–15.90; wingspan 23.40–26.54; fore wing 10.01–11.34. Structure and color. Head black; paraocular area with dense black hairs; central area of clypeus with strong median carina; apical margin of clypeus with medioapical tubercle and two lateral tubercles; subtriangular supraclypeal area with sparse punctures, apical and median area with strong carina; mandible with two stout apical teeth at apex and three small teeth basally, without cutting edge; outer surface of mandible minutely roughened with long black hairs; labrum rectangular, with surface minutely roughened and brimmed with erected long brown hairs along margins, conspicuously at apex; gena with sparse punctures; almost bare vertex with sparse punctures, ID shorter than OD, ID/OD = 0.50 ± 0.01; antennae with ten flagella, first flagellomere wider than long but shorter than the second; body parallel-sided, thorax covers with white hairs except central area of mesoscutum and scutellum; mesoscutum and lower part of mesepisternum with coarsely striate puncture pattern; procoxal base with conspicuous small carina, covered with sparse white hairs; pro- and mesotibiae with two spines at apices; metatarsus with one small spine at apex; protarsus covers with dense brown hairs; meso- and metatarsus cover with dense brown hairs on outer side, with dense ferruginous hairs inner side; wing brown with dark brown vein; T1 covers with sparse ferruginous hairs, with sparse punctures; T2–T5 cover with dense ferruginous hairs, dense punctures on pregradular area, sparse punctures on marginal zone; T6 covers with dense ferruginous hairs, with sparse punctures and round shape at apex; scopa ferruginous except the basal area with white.

#### Literature records.

Laos. Houaphan (Ascher and Pickering 2020); Malaysia. Kelantan (Ascher and Pickering 2020); Myanmar. Kayin, Tangdong, Tenasserim. (Friese 1903; Ascher and Pickering 2020). Also recorded based on images on iNaturalist on two occasions from Huai Mae Priang, Kaeng Krachan District, Phetchaburi Province (credit: djhiker 2016; pam-piombino 2017).

#### Material examined.

Thailand. Chiang Mai Province: 1♀, Chom Thong district, Doi Inthanon Nat. P., Wildfire Control Station, 18°37'7.0590"N, 98°36'29.7606"E, Alt. 779 m, 20-VII-2015, coll. Warrit et al. (leg. NC and NW). Phitsanulok Province: 2♀, Phuhinrongkla Nat. P., 16°59'49.3008"N, 101°0'40.6772"E, Alt. 1303 m, 17-VI-2017, coll. N. Warrit et al. (leg. NC and NW).

#### Floral record.

Specimens from Phitsanulok province were collected on *Craspedolobium unijugum* (Gagnepain) Z. Wei & Pedley at Phuhinrongkla National Park along with *M. umbripennis* and Megachile (Creightonella) fraterna Smith, 1853.

#### Comments.

In the field, *M. impressa* can be confused with Megachile (Creightonella) fraterna since that species also has a black head and ferruginous hairs on abdomen. However, *M. fraterna* can be easily discriminated by characters in the mandible and cutting edge between interspaces.

#### Remarks.

Known sites for this species are in the highlands.

### Megachile (Callomegachile) memecylonae

Taxon classificationAnimaliaHymenopteraMegachilidae

(Engel, 2011)

08F2617F-B8D2-57DB-B21E-B76E06D061F6

Figs 13 , 14

Chalicodoma (Alocanthedon) memecylonae Engel, 2011: 63–67; Male holotype (NHMUK, examined), Penang: Batu Ferringgi, Malaysia.

#### Diagnosis.

Megachile (Callomegachile) memecylonae (Engel, 2011) superficially resembles M. (Callomegachile) atratiformis (Meade-Waldo, 1914), in overall appearance and size: large body size (18–19 mm); black body covered with black hairs throughout; yellow wings. Female is easily distinguished by mesoscutum with distinctly transverse wrinkle pattern on disc, posteriorly also with well separated punctures (Engel and Gonzalez 2011). Male can be recognized by median cell of forewings without black setae patch; juxtamandibular flange present (Fig. 14d); basitarsi of pro legs with hook shape (Fig. 14e).

**Figure 13.**
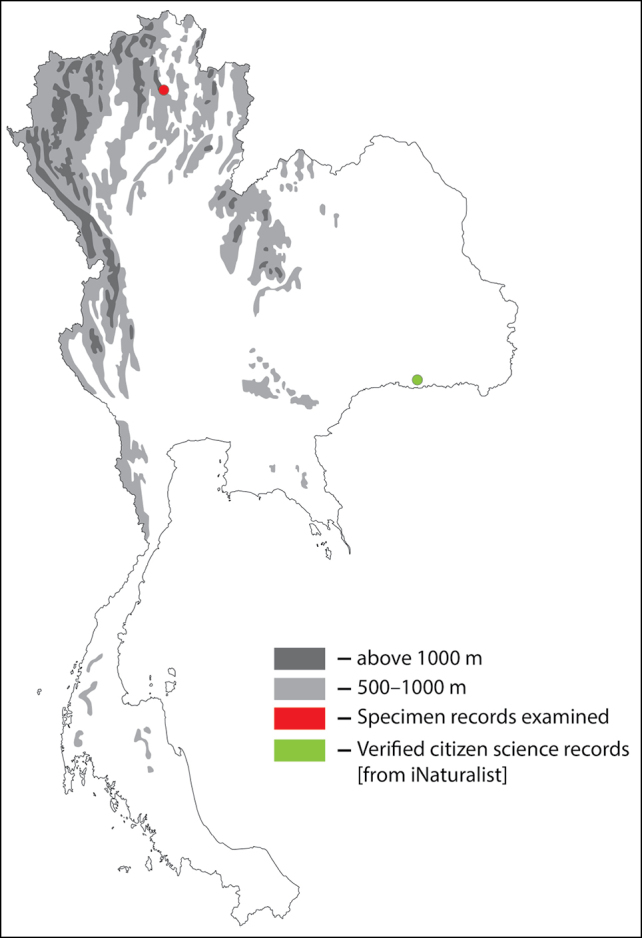
Thai distribution of Megachile (Callomegachile) memecylonae.

**Figure 14.**
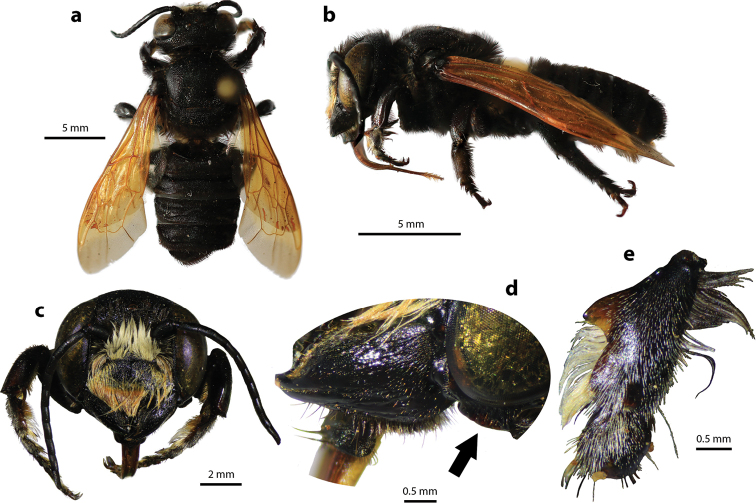
Megachile (Callomegachile) memecylonae (Engel, 2011), male **a** dorsal view **b** lateral view **c** frontal view **d** juxtamandibular flange **e** basitarsi of front leg.

#### Literature records.

Malaysia. Kuala Lumpur, Pahang, Pangkor Island, Penang, Perak, Selangor (Engel and Gonzalez 2011). A photograph on iNaturalist from Buachet, Surin Province may pertain to this species but visual identification in this group is difficult (credit: janescan 2018).

#### Material examined.

Male holotype. Malaysia. “Holotype; B.M. TYPE HYM 17a. 3179; memecylonae, Batu Feringgi; 17 xi 1963 HTP. ⌀147; H.T. Pagden Coll. B.M. 1971–46; Malaya, Penang, Batu Feringgi, 17 Nov 1963, H T Pagden; Holotype, *Chalicodoma memecylonae* Michael S. Engel; NHMUK 013380270”; Thailand. Phayao Province: 1♂, Mueang district, Mae Ka subdistrict, Phayao University, 01-VI-2012, coll. Warrit et al. (leg. NW).

#### Remarks.

This species was described from Peninsular Malaysia. It is remarkable that the first and only Thai specimen record is from Phayao Province in northern Thailand.

### Megachile (Callomegachile) monticola

Taxon classificationAnimaliaHymenopteraMegachilidae

Smith, 1853

39C1453B-DEBE-5F44-9D66-D78D8A89CECB

Figs 15 , 16

Megachile monticola Smith, 1853: 179. Female syntype (NHMUK, examined).
Megachile felderi Radoszkowski, 1882: 79.
Megachile rhinoceros Mocsáry, 1892: 131.
Megachile samson Cameron, 1897: 128.
Megachile koshunensis Strand, 1913: 60.
Chalicodoma (Eumegachilana) monticola : Michener, 1965: 192.

#### Diagnosis.

Female can be recognized by its black large body size (20–26 mm); mesosoma and T1 covered with fulvous hairs (Fig. 16a); base of clypeus with large protruding tubercle (Fig. 16b); mandibles elongate with three teeth and small tubercle at base; labrum oblong with lateral impression (Fig. 16c); black scopa.

**Figure 15.**
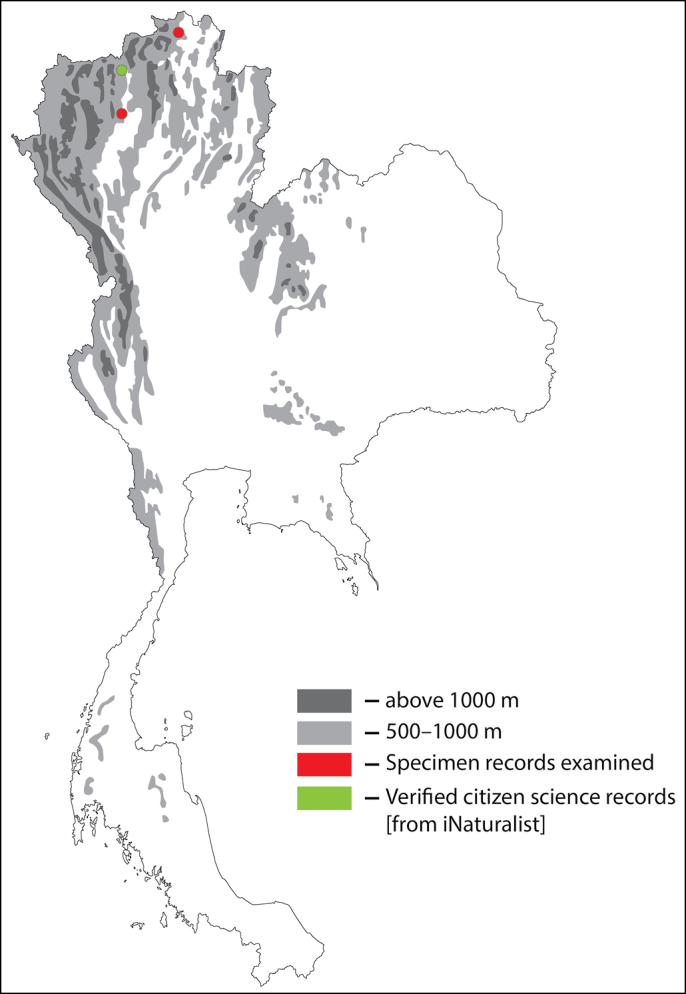
Thai distribution of Megachile (Callomegachile) monticola.

**Figure 16.**
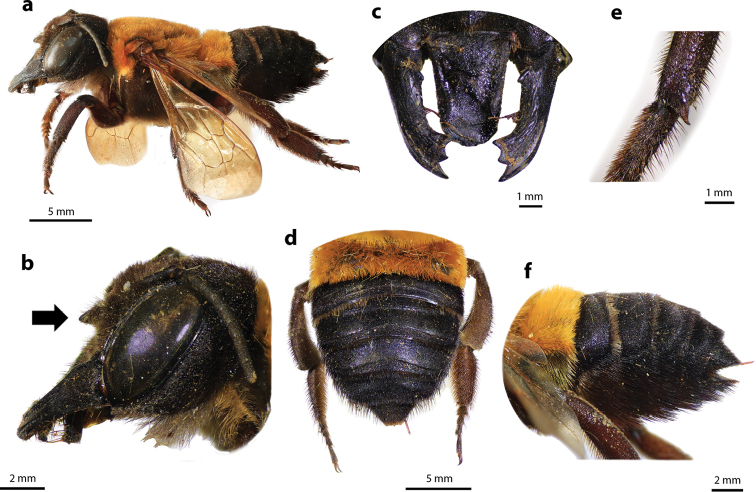
Megachile (Callomegachile) monticola Smith, 1853, female **a** lateral view **b** protruding tubercle at base of clypeus (arrow) **c** frontal view of mandible and labrum **d** dorsal view of metasoma **e** spine at apical tibia **f** lateral view of metasoma and scopa.

#### Literature records.

Bangladesh. Sylhet (Smith 1853; Bingham 1897); China. Anhui, Fujian, Shanghai (Smith 1853); Hong Kong.; India. Assam, Sikkim (Bingham 1897); Indonesia. Lombien Island (Ascher and Pickering 2020); Japan. Okinawa, Uragami (Ascher and Pickering 2020); Myanmar. Tenasserim (Bingham 1897); Taiwan. Taihoku-shu (Ascher and Pickering 2020); Vietnam. Thua Thien-Hue (Ascher and Pickering 2020). Also reported from Chiang Mai Province on iNaturalist (credit: entomokot 2019).

#### Material examined.

Female syntype. “Type; B.M. TYPE HYM. 17. a. 2155; Sylhet, 4[?]7 51; *Megachile monticola*, TYPE, Sm.; monticola Type Sm.; NHMUK 013379845”; Thailand. Chiang Mai Province: 1♀, Mueang district, Chang Phuag subdistrict, Nong Hoe, 17-VII-1996, coll. Adul (leg. NC and NW); 1♀, longan plantation, 10-II-2009, coll. Paveenun (leg. NC and NW); 1♀, 20-IX-1985, coll. Sumrid (leg. NC and NW). Chiang Rai Province: 1♀, Mae Chan district, Mae Chan subdistrict, 14-VIII-1960, coll. unknown (leg. NC and NW).

### Megachile (Callomegachile) odontophora

Taxon classificationAnimaliaHymenopteraMegachilidae

(Engel, 2011)

F57F3717-9BCA-5BC9-B850-8A57C1164059

Figs 17 , 18

Chalicodoma (Alocanthedon) odontophorum Engel, 2011: 55–60; Female paratype (NHMUK, examined) Nakhon Ratchasima, Thailand.

#### Diagnosis.

Female superficially resembles M. (Callomegachile) atratiformis (Meade-Waldo, 1914) in overall appearance and size: large body size (20–24 mm); black body covered with black hairs throughout; mesoscutum with weak transverse wrinkle pattern on disc, also posteriorly with weakly transverse wrinkle pattern; yellow wings (except apical margin of clypeus with median tubercle (Fig. 18e); mandibles four teeth with three stout apical teeth at apex and small tooth basally; labrum oblong with pointed apical margin and two lateral teeth (Fig. 18d)). Male can be easily recognized by clypeus covered with densely long hairs; forewings with black setae patch on medial cell; modified front tarsi (Engel and Gonzalez 2011).

**Figure 17.**
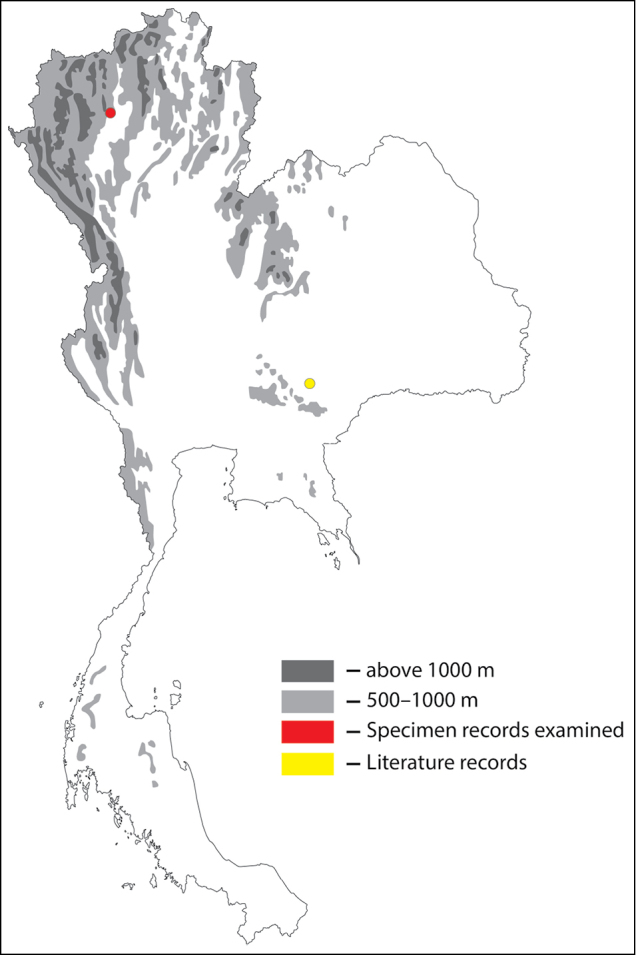
Thai distribution of Megachile (Callomegachile) odontophora.

**Figure 18.**
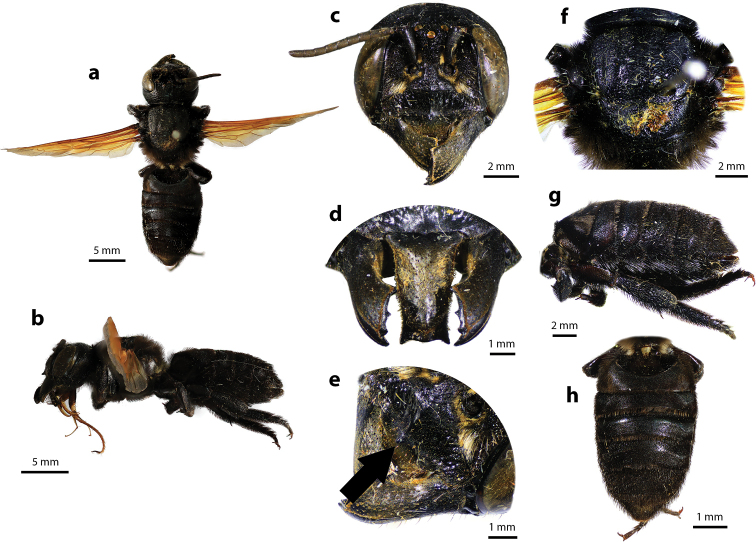
Megachile (Callomegachile) odontophora (Engel, 2011), female **a** dorsal view **b** lateral view **c** frontal view **d** frontal view of mandible and labrum **e** tubercle at clypeal margin (arrow) **f** dorsal view of mesoscutum **g** lateral view of metasoma and scopa **h** dorsal view of metasoma.

#### Literature records.

Myanmar. Thaungyin valley (Middle Tenasserim) (Engel and Gonzalez 2011); Thailand. Nakhon Ratchasima (Engel and Gonzalez 2011).

#### Material examined.

Type material. Female paratype. Myanmar. “Middle Tenasserim; Thaungyin [= Moei River] Valley, 5/93, C.T. Bingham; Col. C.T. Bingham 96–30; Paratype, *Chalicodoma odontophorum* Michael S. Engel; NHMUK 013380271”; Thailand. Chiang Mai Province: 1♀, 13-VII-2006, coll. M. Rungrote (leg. NC and NW).

#### Floral record.

Engel and Gonzalez (2011) noted the species was captured on *Sindora siamensis* Teijsman & Miquel.

### Megachile (Callomegachile) ornata

Taxon classificationAnimaliaHymenopteraMegachilidae

Smith, 1853

91721A77-C422-537E-A0C5-92898CDD2C29

Figs 19 , 20

Megachile ornata Smith, 1853: 183; female syntype (NHMUK, examined) Indonesia.
Megachile miniata Bingham, 1896: 199.
Megachile ruficorbis Cockerell, 1927: 6.

#### Diagnosis.

Female can be recognized by its black large body size (17–19 mm); T1–T4 covered with black hairs; T2 with small patch of brick-red hairs laterally; T5–T6 covered with pale light yellow hairs (Fig. 20a); mandible three teeth (Fig. 20b); second spine of pro- and mesotibiae bifurcate (Fig. 20c); metatibiae with spine at apex (Fig. 20d); brick-red scopa.

**Figure 19.**
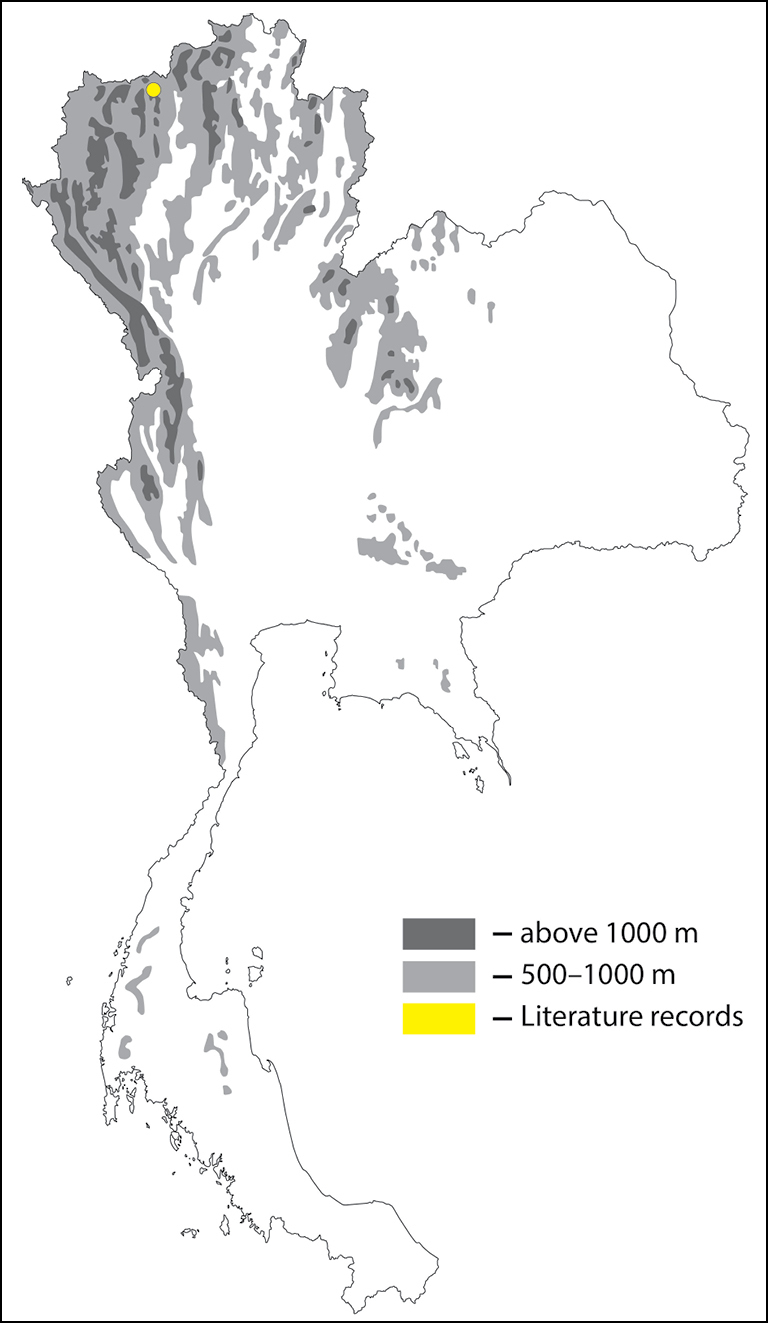
Thai distribution of Megachile (Callomegachile) ornata.

**Figure 20.**
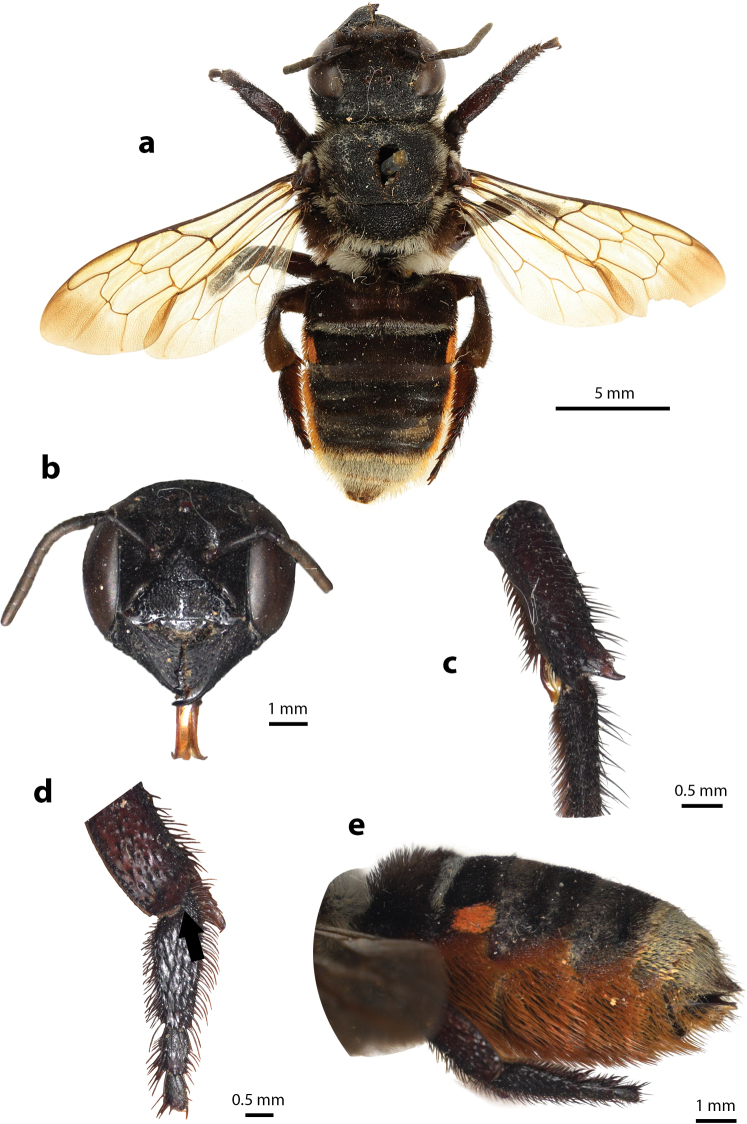
Megachile (Callomegachile) ornata Smith, 1853, female (NHMUK 013379840) **a** dorsal view **b** frontal view **c** spine at front tibia **d** spine at hind tibia **e** lateral view of mesosoma and scopa.

#### Literature records.

Brunei. (Ascher and Pickering 2020); India. (Tadauchi and Tasen 2009); Indonesia. Borneo, Sumatra: including Deli (Kirby 1894; Meade-Waldo 1912; Tadauchi and Tasen 2009; Ascher et al. 2016); Malaysia. Kuala Lumpur, Negeri Sembilan, Pahang, Sabah, Sarawak, Selangor (Cockerell 1927a; Ascher et al. 2016; Ascher and Pickering 2020); Myanmar. (Cockerell 1927a); Nepal. (Tadauchi and Tasen 2009; Ascher and Pickering 2020); Singapore. (Ascher et al. 2016); Thailand. Chiang Mai (Tadauchi and Tasen 2009).

#### Material examined.

Female syntype. Indonesia. “56 43; Locality unknown, pre-1853, Exchanged unit, Mr. Baly, B.M. 1856–43; Syntype, female, *Megachile ornata* Smith, F., 1853:183, det. D. Notton 2018 (ICZN Rec. 73F); B.M. TYPE HYM. 17a. 3215; NHMUK 013379840”.

#### Comments.

Trunz et al. 2016’s phylogenetic analysis suggested *M. ornata* belongs to a distinct lineage of *Callomegachile* sensu lato.

#### Floral records.

Megachile (Callomegachile) ornata was captured on *Grammatophyllum speciosum* Blume (Ascher et al. 2016).

### Megachile (Callomegachile) parornata

Taxon classificationAnimaliaHymenopteraMegachilidae

Chatthanabun, Warrit and Ascher nom. nov.

E50156CC-28AF-5A37-AC8C-C6925CDD3FB5

Figs 21 , 22

Megachile gigas Wu, 2005: 159; preoccupied (junior primary homonym, not Megachile gigas Schrottky, 1908, Brazil). Female holotype (erroneously described as male) (IZB, examined) Xiaomengyang, Xishuangbanna, Yunnan, China.

#### Diagnosis.

Female superficially resembles M. (Callomegachile) ornata Smith, 1853 except T1 covered with black hairs; T2–T5 covered with brick-red hairs; T6 covered with pale yellow hairs (Fig. 22g, h); scopa brick-red.

**Figure 21.**
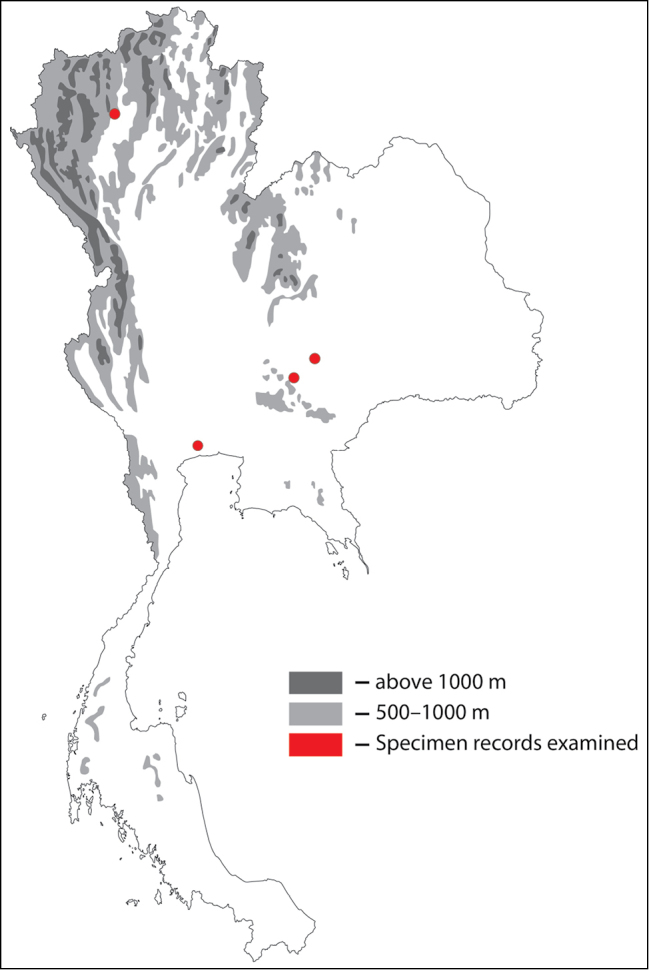
Thai distribution of Megachile (Callomegachile) parornata.

**Figure 22.**
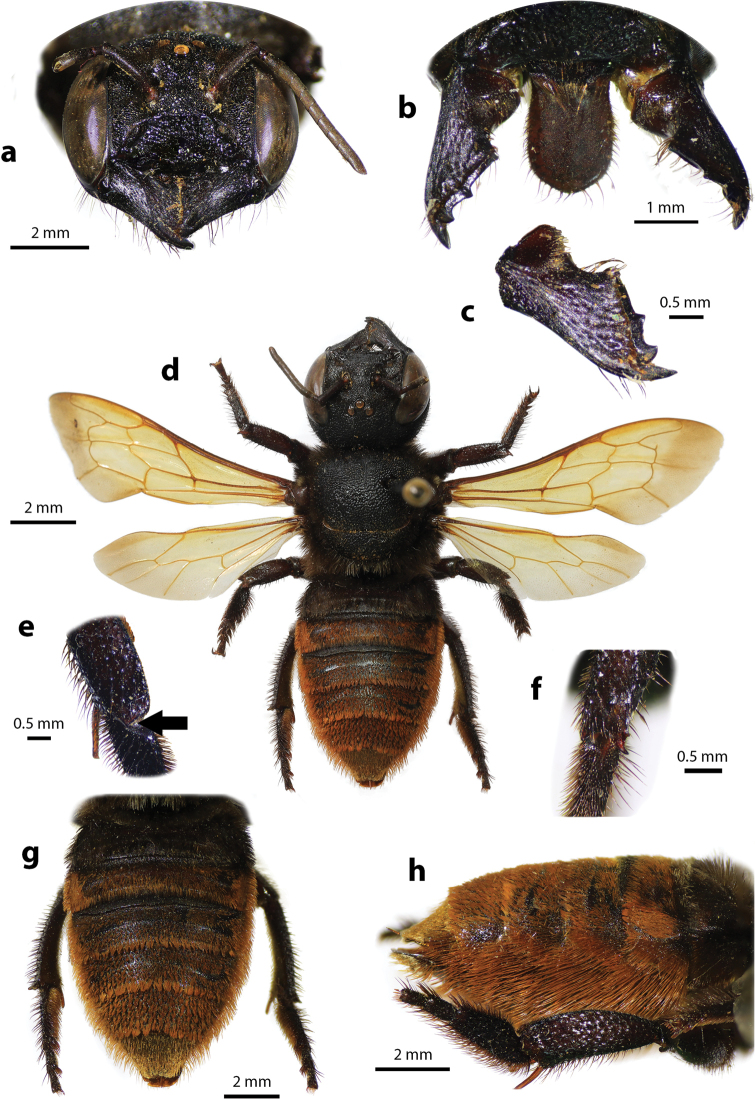
Megachile (Callomegachile) parornata Chatthanabun, Warrit and Ascher, nom. nov., female **a** frontal view **b** labrum and mandibles **c** mandible **d** dorsal view **e** spine on hind tibia **f** spine on front tibia **g** dorsal view of metasoma **h** lateral view of metasoma and scopa.

#### Redescription.

***Female*.** Length. Total body length 20.00–21.50; wingspan 35.55–38.24; forewing 14.84–16.13. Structure and color. Head black; ventral margin of paraocular area with brown hairs; clypeus trapezoid with rough surface and sparse punctures; supraclypeal area convex and subtriangular with rough surface and sparse punctures; mandible with three teeth, without cutting edge; outer surface of mandible minutely roughened with long brown hairs; labrum length twice as long as wide with round apex, surface minutely convex and rough with erect long brown hairs at apex; gena with sparse punctures; bare vertex with sparse punctures, ID shorter than OD, ID/OD = 0.28 ± 0.01; antennae with eleven flagella, first flagellomere wider than long, shorter than the second; body parallel-sided; scutum and scutellum hairless except anterior margin of scutum with brown hairs; lower part of metathorax with white hairs; scutum and lower part of mesepisternum with coarsely striate puncture pattern; procoxa covers with brown hairs; pro- and mesotibiae with two apical spines, mesotibial spine bifurcate; apex of metatibiae with spine at apex; pro- and mesotarsus with short brown hairs; metatarsus with dense short fulvous hairs inner side and short brown hairs outer side; forewing length yellow hyaline with yellowish-brown vein; T1 covers with black hairs; T2–T5 cover with brick-red hairs; T6 covers with pale yellow hairs, round apex; scopa fulvous-red.

***Male*.** Unknown.

#### Literature records.

China. Yunnan (Wu 2005); Vietnam. Hoa-Binh, Vinh-Quang (Wu 2005).

#### Material examined.

Female holotype (erroneously described as male). China. “Yunnan, Xishuangbanna, Xiaomengyang (22°N, 100.8°E), 850 m; 1957. IX. 6, collected by Zang Ling-Chao; Holotype; female, Megachile (Callomegachile) gigas Wu, 2005; det. Y. R. Wu; IOZ(E) 210406”; Thailand (new record). Bangkok Province: 1♀, Dusit, 8-IX-1968, coll. Patchanee (leg. NC and NW). Chiang Mai Province: 1♀, Meuang district, Faculty of Agriculture, 4-VIII-1981, coll. Vijit (leg. NC and NW); 1♀, Meuang district, Chiang Mai University, 6-VIII-1981, coll. Sumrid (leg. NC and NW). Nakhon Ratchasima Province: 1♀, 30-VI-1962, coll. unknown (leg. NC and NW); 1♀, Faculty of Forestry, 10-VII-1968, coll. K. Vajropala (leg. NC and NW).

#### Etymology.

The species name refers to the close resemblance to *M. ornata*.

#### Remarks.

The wide published distribution of *M. ornata* from Nepal to the Indonesian Archipelago raises a question regarding whether this is a single species or a species complex. Since the type material of *M. ornata* was collected from Sumatra, Indonesia, specimens reported under this name from other mainland Asian countries should be re-examined. Furthermore, whereas no additional specimens of *M. parornata* were collected and deposited in NHMCU-BSRU and CMU since 1968, Tadauchi and Tasen (2009) recorded one specimens of *M. ornata* from Chiang Mai province (not seen by us). Until, male specimens of both *M. parornata* and *M. ornata* are collected and studied, we are proposing *M. parornata* to be a separate species from *M. ornata*.

### Megachile (Callomegachile) tuberculata

Taxon classificationAnimaliaHymenopteraMegachilidae

Smith, 1858

724DFBBA-BC94-56C9-AF62-E5BD47755CA7

Figs 23 , 24

Megachile tuberculata Smith, 1858: 46. Female syntype (NHMUK, examined) Borneo, Malaysia.
Megachile longipalpis Radoszkowski, 1882: 78 [doubtful synonymy].
Chalicodoma (Eumegachilana) tuberculatum : Michener, 1965: 192.

#### Diagnosis.

Female can be recognized by its large body size (21–24 mm); black body covered with black hairs throughout (Fig. 24a); base of clypeus with large protruding tubercle; mandibles elongate with three teeth and small tubercle at base (Fig. 24c); yellow wings; black scopa.

**Figure 23.**
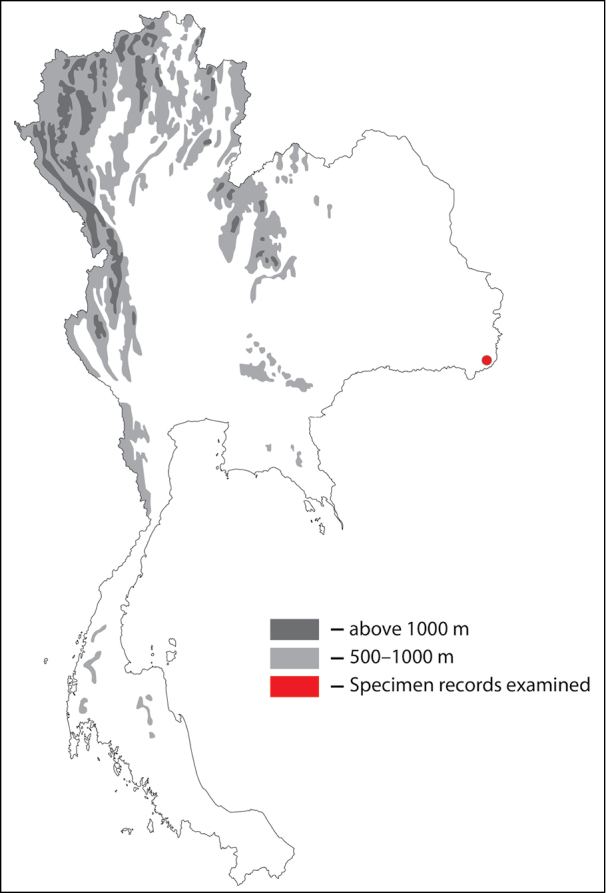
Thai distribution of Megachile (Callomegachile) tuberculata.

**Figure 24.**
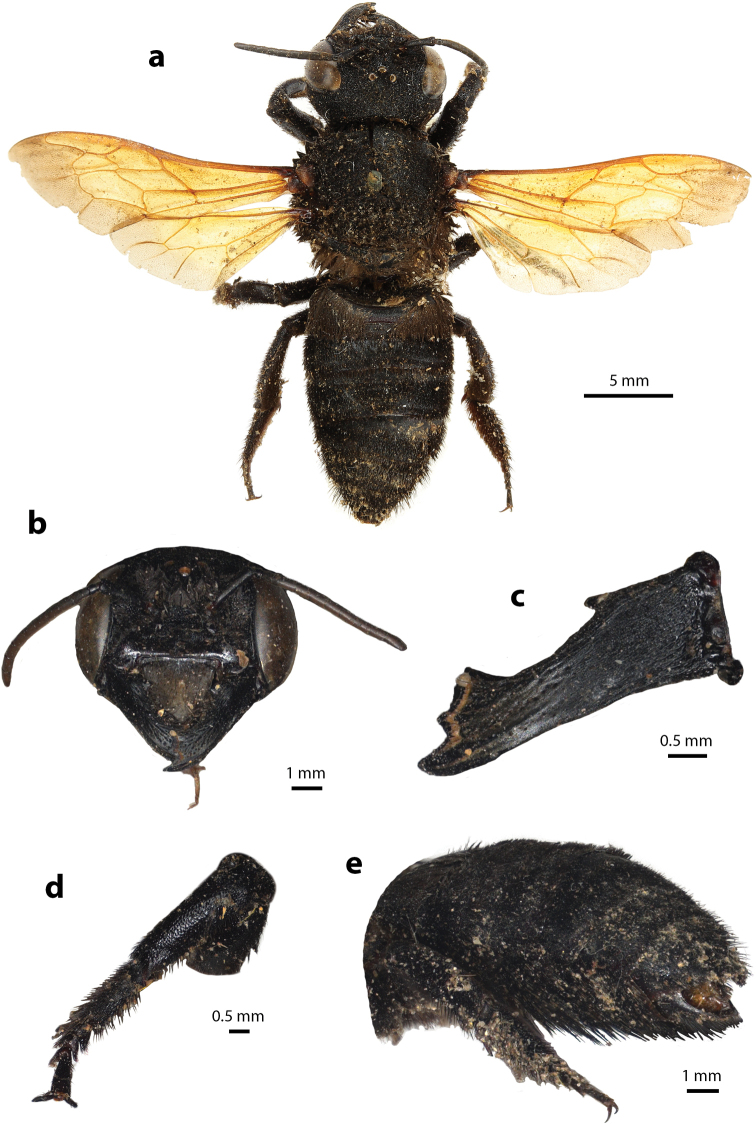
Megachile (Callomegachile) tuberculata Smith, 1858, female (NHMUK 013379846) **a** dorsal view **b** frontal view **c** lateral view of mandible **d** front leg **e** lateral view of metasoma and scopa.

#### Literature records.

India. Sikkim (Ascher et al. 2016); Indonesia. Java, Sumatera Barat (Ascher et al. 2016); Myanmar. Mergui Archipelago, Bago Yoma (Bingham 1890, 1897); Malaysia. Johor, Negeri Sembilan, Pahang, Perak, Sabah, Sarawak, Selangor, Terengganu, (Smith 1858; Bingham 1890, 1897; Dover 1929; Ascher et al. 2016); Philippines. (The Philippines record based on the type of *M. longipalpis* Radoszkowski, 1882 is considerably outside the verified range for this species as presently understood, so this synonymy should be re-examined); Singapore. (Bingham 1897; Ascher et al. 2016).

#### Material examined.

Female syntype. Malaysia. “Type; B.M. TYPE HYM. 17a. 2840; SAR.; *Megachile tuberculata* Sm. 1857 not 1879; NHMUK 013379846”; Thailand (new record). Ubon Ratchathani Province: 1♀, Na Chaluai district, Phu Jong Nayoy Nat. P., Phalan Pa Chad, 14°26'7.8066"N, 105°15'34.7394"E, Alt. 247.17 m, 6-XI-2019, coll. Traiyasut et al. (leg. NC and NW).

#### Floral records.

Megachile (Callomegachile) tuberculata was photographed visiting and collecting pollens from *Psophocarpus tetragonolobus* (L.) D.C. (Soh et al. 2017), also captured on *Grammatophyllum speciosum* Blume (Ascher et al. 2016).

#### Comments.

The single Thai record is from far to the northeast of the Sundaic Region.

### Megachile (Callomegachile) umbripennis

Taxon classificationAnimaliaHymenopteraMegachilidae

Smith, 1853

A707227D-ABCA-5A79-83E7-8EA425C857DA

**Figure 25.**
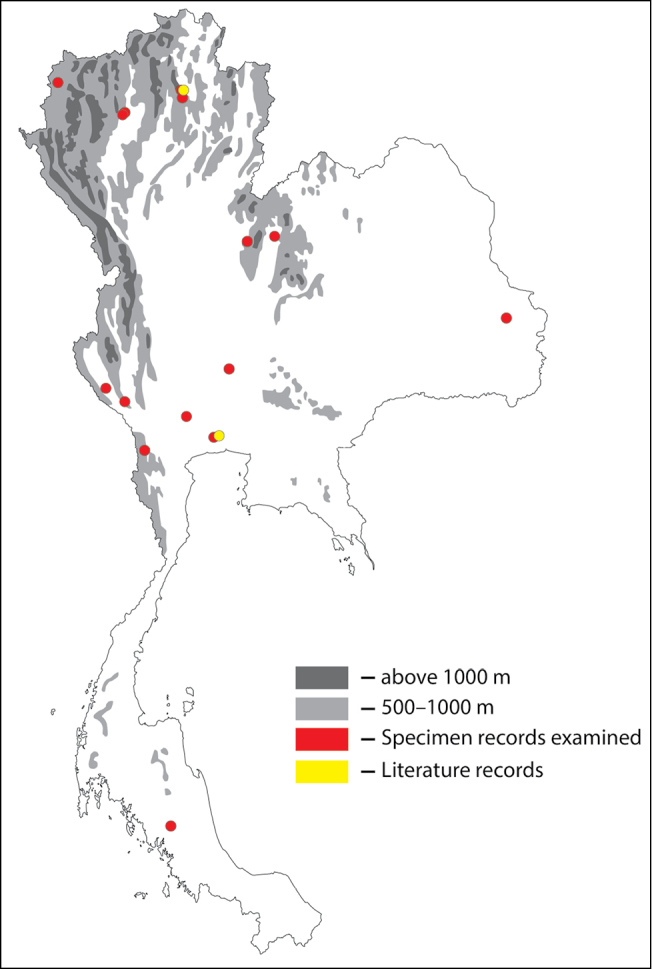
Thai distribution of Megachile (Callomegachile) umbripennis.

**Figure 26.**
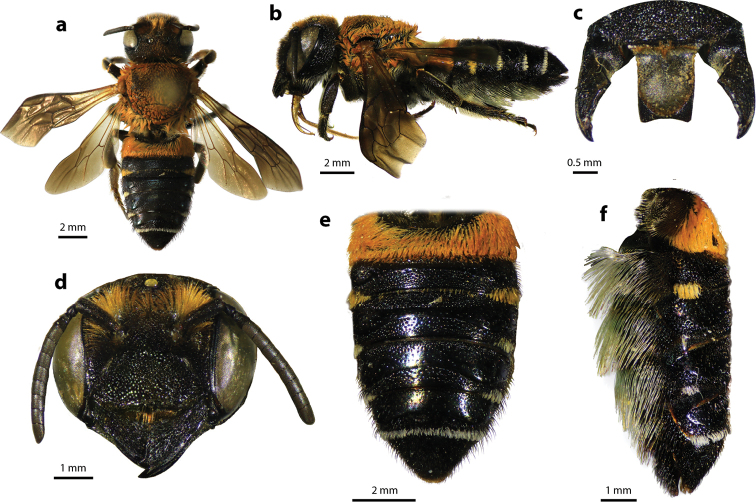
Megachile (Callomegachile) umbripennis Smith, 1853, female **a** dorsal view **b** lateral view **c** frontal view of mandible and labrum **d** frontal view **e** dorsal view of metasoma **f** lateral view of metasoma and scopa.

**Figure 27.**
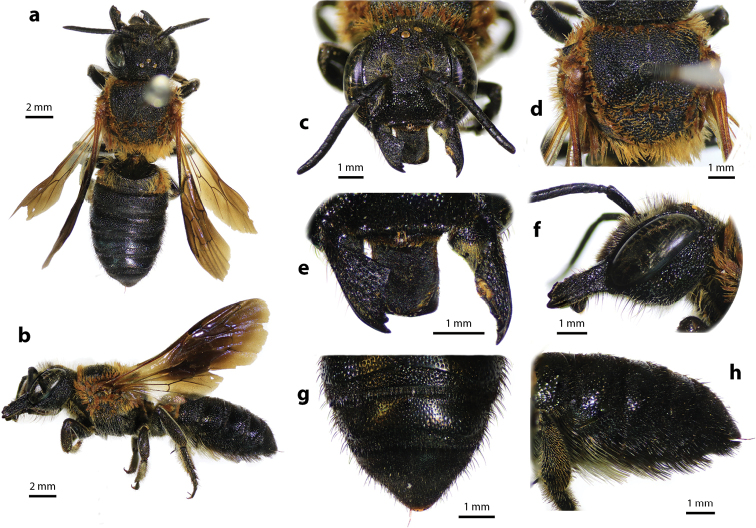
Megachile (Callomegachile) umbripennis Smith, 1853 (BSRU AA-4620), female **a** dorsal view **b** lateral view **c** frontal view **d** dorsal view of mesoscutum **e** clypeus and mandible **f** lateral view of mandible **g**T6**h** lateral view of metasoma showing scopa.

**Figure 28.**
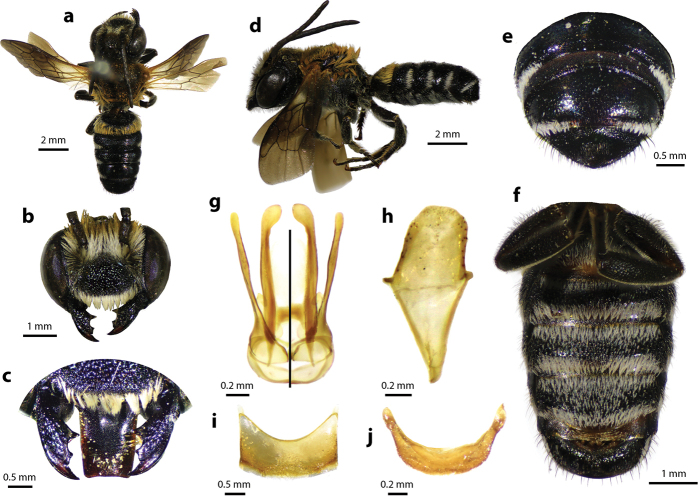
Megachile (Callomegachile) umbripennis Smith, 1853, male **a** dorsal view **b** frontal view **c** frontal view of mandible and labrum **d** lateral view **e** frontal view of T7 **f** ventral view of metasomal sterna **g** dorsal (left) and ventral (right) views of penis **h** dorsal view of S8 **i** S5 **j** T7.

**Figures 29.**
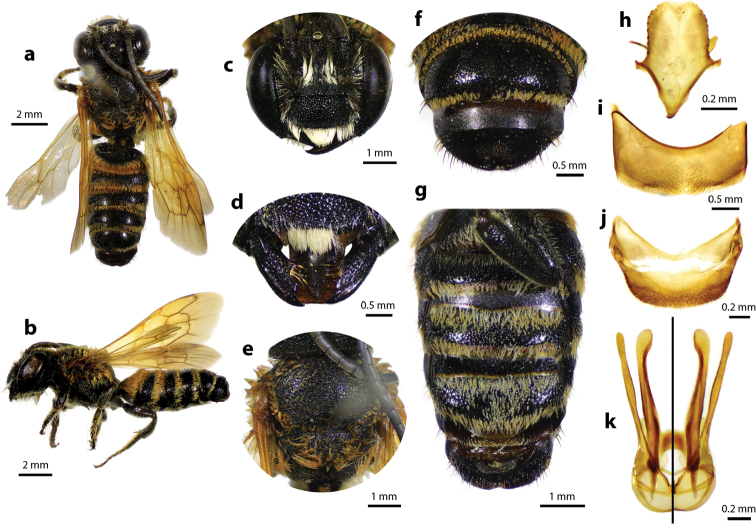
Megachile (Callomegachile) umbripennis Smith, 1853 (BSRU AA-3654, BSRU AA-3662 and KKIC-02), male **a** dorsal view **b** lateral view **c** frontal view **d** clypeus and mandible **e** dorsal view of mesoscutum **f**T6**g** ventral view of metasomal sterna showing scopa dorsal view of metasomal sterna **h** dorsal view of S8 **i** S5 **j** T7 **k** dorsal (left) and ventral (right) views of penis.

Figs 25 , 26 , 27 , 28 , 29

Megachile umbripennis Smith, 1853: 175. Female syntype (NHMUK, examined) Nepal [as Nepaul].
Megachile schauinslandi Alfken, 1898: 340.
Megachile domesticum Perkins, 1899: 114, nomen nudum.
Megachile umbripennis var atriventris Friese, 1903: 357.
Megachile aureobasis Cockerell, 1919: 198.
Megachile (Eumegachile) umbripennis : Krombein, 1950: 125.
Chalicodoma (Callomegachile) umbripennis : Michener, 1965: 191.

#### Diagnosis.

Female superficially resembles leafcutter bee M. (Aethomegachile) laticeps Smith, 1853, in terms of its overall appearance and size: mesosoma and propodeal triangle with fulvous hairs, except vertex and disc area of mesoscutum covered with fulvous hairs (Fig. 26a); T2–T4 with tuft fulvous hairs laterally; T5 with white hair band (Fig. 26e). Male is similar to female except apical margin of clypeus covered with hairs; mandibles three teeth (Fig. 28b, c).

#### Literature records.

American Samoa; China. Aitutaki, Canton, Fujian, Guandong, Sichuan, Yunnan (Ascher et al. 2016); Fiji. (Davies et al. 2013; Ascher et al. 2016); French Polynesia. Mo’orea, Society Islands, Tahiti (Ascher et al. 2016); Hong Kong. (Ascher et al. 2016); India. Chandigarh, Haryana, Sikkim, Tamil Nadu (Bingham 1897; Ascher et al. 2016); Laos. Vientiane (Ascher et al. 2016); Malaysia. Kelantan, Kuala Lumpur, Pahang, Sarawak, Selangor (Smith 1858; Ascher et al. 2016); Mariana Islands. San Vicente; Myanmar. Tenasserim (Bingham 1897; Ascher et al. 2016); Nepal. (Smith 1853, 1858; Bingham 1897; Ascher et al. 2016); New Caledonia. (Ascher et al. 2016); Northern Mariana Islands. (Ascher et al. 2016); Singapore. (Ascher et al. 2016); Sri Lanka. Matale (Ascher et al. 2016); Cook Islands. Aitutaki, Rarotonga, Mangaia (Kuhlmann 2006; Ascher et al. 2016); Tonga. (Ascher et al. 2016); USA. Florida, Hawaii, Kauai, Midway, Maui, Molokai, Oahu (Ascher et al. 2016); Vietnam. Ha Giang (Ascher and Pickering 2020).

#### Material examined.

Female syntype. Nepal. “Type; B.M. TYPE HYM. 17. a. 2160; Hardwicke Bequest; Nepal [as Nepaul]; umbripennis, Type, Sm.; *Megachile umbripennis* TYPE. Sm.; NHMUK 013380267”; Thailand. Chiang Mai Province: 1♀, 16♂, Mueang district, Mae Hia subdistrict, Mae Hia Agricultural Research, Demonstrative and Training Center, 18°45'51.1272"N, 98°55'39.6192"E, Alt. 232 m, 19-VII-2015, coll. Warrit et al. (leg. NC and NW). Kanchanaburi Province: 1♀, 19♂, Sai Yok district, Wang Krachae subdistrict, 14°9'56.7678"N, 98°55'39.6192"E, Alt. 232 m, 24-VI-2016, coll. Warrit et al. (leg. NC and NW); 8♂, Sai Yok district, Wang Krachae subdistrict, 14°11'6.5724"N, 99°3'6.9258"E, Alt. 102.30 m, 24-VI-2016, coll. Warrit et al. (leg. NC and NW); 1♀, Sai Yok district, Sai Yok Yai National Park, 14°27'16.4118"N, 98°51'40.4928"E, Alt. 49.17 m, 24-VI-2016, coll. Warrit et al. (leg. NC and NW). Mae Hong Son Province: 1♀, Pang Ung, Pang Tong Under Royal Forest Park 2, 19°29'58.3008"N, 97°54'42.1014"E, Alt. 1,164 m, 10-XII-2015, coll. Warrit et al. (leg. NC and NW). Nakhon Pathom Province: 1♀, Kamphaeng Saen district, Kamphaeng Saen subdistrict, Kasetsart University, Kamphaeng Saen Campus, 22-II-2000, coll. Sokroh (leg. NC and NW); 1♀, Kamphaeng Saen district, Kamphaeng Saen subdistrict, Kasetsart University, Kamphaeng Saen Campus, 22-II-2003, coll. Kittisak (leg. NC and NW); 2♂, Kamphaeng Saen district, 13°44'58.3908"N, 99°52'33.1242"E, Alt. 14 m, 10-VII-2015, coll. Warrit et al. (leg. NC and NW). Phayao Province: 1♀, 4♂, Mueang district, Maeka subdistrict, Phayao University, 01-VI-2012, coll. Warrit et al. (leg. NC and NW); 1♀, 28-I-2014, coll. S. Yutham (leg. NC and NW). Phetchabun Province: 2♂, Lomsak district, Bungkla subdistrict, 19-X-2009, coll. K. Attasopa & P. Phukphume (leg. NC and NW); 1♀, 2♂, Lomsak district, Bungkla subdistrict, 20-X-2009, coll. K. Attasopa & P. Phukphume (leg. NC and NW); 1♀, Lomsak district, Bungkla subdistrict, 21-X-2009, coll. K. Attasopa & P. Phukphume (leg. NC and NW). Phitsanulok Province: 1♀, Phuhinrongkla Nat. P., Lan hin pum, 16°59'49.8008"N, 101°00'40.6772"E, Alt. 1303 m, VI. 17. 2017., Aerial net, coll N. Warrit et al. (leg. NC and NW). Ratchaburi Province: 1♀, Suan Pheung district, 25-V-2012, coll. N. Warrit (leg. NC and NW). Samut Sakhon Province: 1♀, Banpaew district, 19-VIII-2014, coll. P. Tangtorwongsakul (leg. NC and NW). Suphan Buri Province: 1♂, 9-X-2012, coll. Veerawan (leg. NC and NW). Trang Province: 1♂, Na Yong district, 7°33'8.0892"N, 99°46'33.6072"E, Alt. 24 m, 11-VI-2015, coll. Warrit et al. (leg. NC and NW). Ubon Ratchathani Province: 1♂, Trakan Phuetphon district, Sarin lake view village, 03-VIII-2014, coll. N. Chatthanabun (leg. NC and NW).

#### Comments.

There are some suspect specimens that show variation in both sexes of *M. umbripennis*. One female collected from Phitsanulok province (BSRU AA-4620) shows the following variations: lack of fulvous hairs on disc area of mesoscutum and lack of white hairs on T2–T5 (Fig. 27a–h). Males collected from Chiang Mai province (BSRU AA-3654, BSRU AA-3662) and Suphan Buri province (KKIC-02) have fulvous hairs on T2–T5 instead of white hairs (Fig. 29a–k).

### Megachile (Callomegachile) chiangmaiensis

Taxon classificationAnimaliaHymenopteraMegachilidae

Chatthanabun & Warrit sp. nov.

22978496-AD7C-5904-B0E7-04338E010844

http://zoobank.org/037E992C-1DB2-4BA4-B5EB-6684A3F4374F

Figs 30 , 31

#### Diagnosis.

The species superficially resembles *M. disjuncta* (Fabricius, 1781) in terms of its overall appearance and size: white tuft of hairs on scutellum, propodeum, and first few segments of metasomal terga; however, the prominent apical half-circular impression of clypeus with strong median carina (Fig. 31e) differentiates *M. chiangmaiensis* sp. nov. from the former. Clypeal impression smooth with dense dark hairs at apex. Such a clypeal impression was also present in another species of *Callomegachile*, *M. ramakrishnae* (Cockerell, 1919), a rare bee collected in Tamil Nadu, India, although the impression in *M. ramakrishnae* is more or less shallower and the pattern of the mesosoma and white hairs band on T2–T3 are absent. The apex of the labrum is strongly pointed medially with two lateral teeth (Fig. 31f).

**Figure 30.**
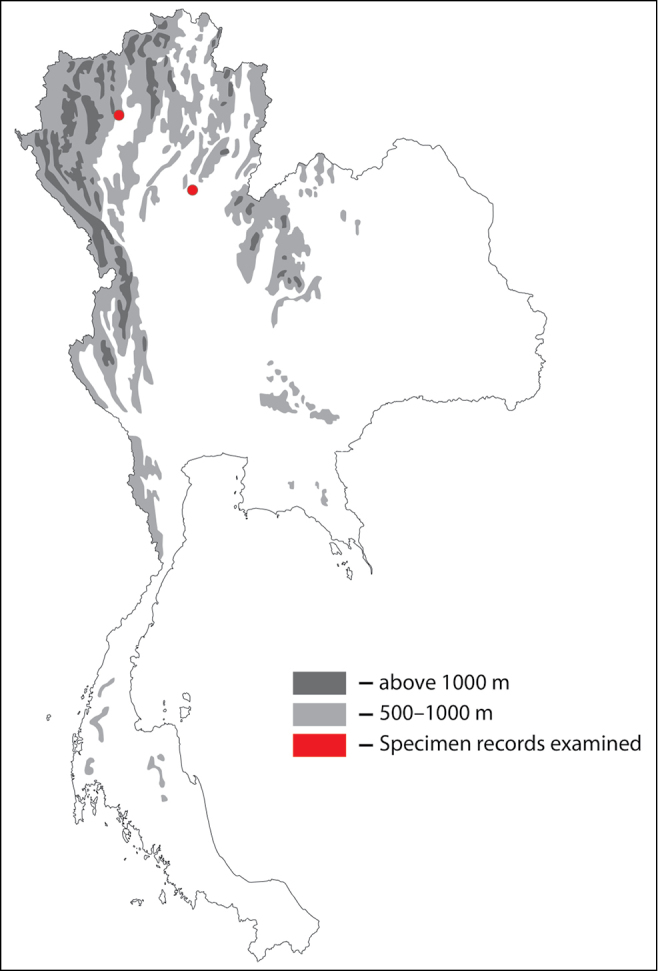
Thai distribution of Megachile (Callomegachile) chiangmaiensis new species.

**Figure 31.**
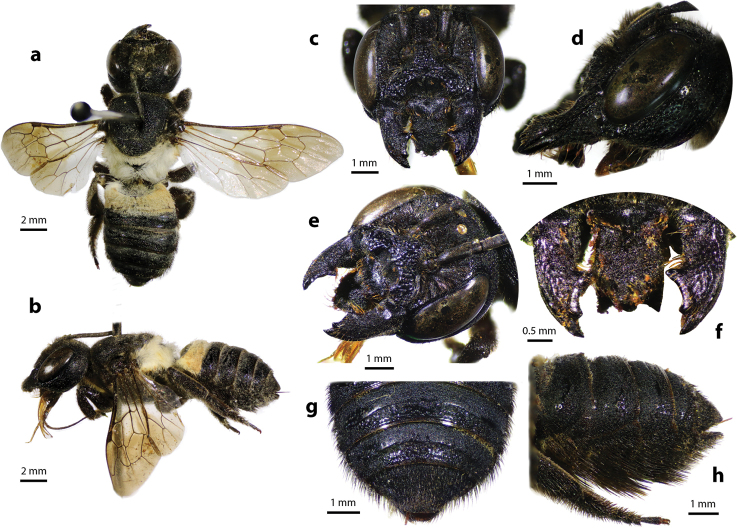
Megachile (Callomegachile) chiangmaiensis Chatthanabun and Warrit, sp. nov., holotype, female **a** dorsal view **b** lateral view **c** frontal view **d** lateral view of mandible **e** oblique view of clypeus **f** clypeus and mandible **g**T6**h** lateral view of metasoma showing scopa.

#### Description.

***Female*.** Length. Total body length 12.57–13.64; wingspan 18.60–22.56; fore wing 9.66 Structure and color. Head black; paraocular area with dense black hairs; clypeus with prominent apical half-circular impression with strong median carina; clypeal impression smooth with dense dark hairs at apex; smooth area of subtriangular supraclypeal with sparse punctures; mandible stout and elongate without cutting edge, mandible with three apical teeth, outer surface minutely roughened with long brown hairs; surface of labrum minutely roughened, apex of labrum medially strongly pointed with two lateral teeth; gena with sparse punctures; sparse punctures on vertex with ID shorter than OD, ID/OD = 0.55 ± 0.06; antennae with ten flagella, first flagellomere wider than long and shorter than the second; body parallel-sided; mesoscutum and lower part of mesepisternum with coarsely striate puncture pattern; mesoscutellum and propodeum with tuft of white hairs; procoxa with small ridge, covered with sparse brown hairs; pro- and mesotibiae with two spines at apices; apex of metatibiae truncate; pro-, meso- and metatarsi with dense brown short hairs; hyaline wings with smoky color at apex and dark brown veins; T1 and pregradular area of T2 covered with tuft of white hairs; T2–T5 with dense punctures on margin and short black hairs on each side; T6 covered with short black hairs, apex round shape; scopa black.

***Male*.** Unknown.

#### Distribution.

Thailand. Chiang Mai and Uttaradit.

#### Material examined.

Female holotype. Thailand. “เชียงใหม่, 11 ก.ย. 56, วิมลชัย [Chiang Mai, 11 September 2013, coll. Vimolchai]” (KKIC-01); Female paratypes. Thailand. “Uttaradit, 8 April 1961, coll. unknown” (3♀, DNP-0002, DNP-0003, DNP-0004).

#### Etymology.

The new species is named after the type locality.

#### Remarks.

*Megachile chiangmaiensis* sp. nov. can be found in the same province as the morphologically similar congeneric species, *M. disjuncta*, although the latter are abundantly collected throughout Thailand. The biology of *M. chiangmaiensis* is unknown.

### Megachile (Carinula)

Taxon classificationAnimaliaHymenopteraMegachilidae

Michener, McGinley, & Danforth, 1994

B4609EE0-6FCE-54A8-AE3A-77DA7B1AD758

Chalicodoma (Carinella) Pasteels, 1965: 447. Type species: Megachile torrida Smith, 1853, by original designation.
Megachile (Carinula) Michener, McGinley, & Danforth, 1994: 174, replacement for Carinella Pasteels, 1965. Type species: Megachile torrida Smith, 1853, autobasic.

#### Diagnosis.

Body size median to small. Female mandible four to five teeth. Clypeus with median carina. Clypeal margin crenulate with five teeth. In males, coxal spine absent, front tarsi simple and carina of T6 extremely reduced.

#### Comments.

*Carinula* is superficially similar to *Callomegachile*, especially striated punctures on mesoscutum and lower part of mesepisternum. Female of *Carinula* can be recognized by the presence of crenulate clypeal margin, whereas male can be recognized by reduced carina on T6.

### Megachile (Carinula) stulta

Taxon classificationAnimaliaHymenopteraMegachilidae

Bingham, 1897

8FA40DBF-67C7-5F54-B30C-779D4085A442

Figs 32 , 33

Megachile stulta Bingham, 1897: 476; Female syntype (NHMUK, examined) Sikkim, India.

#### Diagnosis.

Female can be recognized by its medium to small body size (7.83–10.39 mm); rough clypeus with median carina, apical margin crenulate (Fig. 33b); mandibles four teeth with two stout apical teeth at apex and two small teeth basally; labrum rectangular (Fig. 33c); metasoma covered with ferruginous hairs (Fig. 33e); scopa ferruginous except white basal area (Fig. 33f).

**Figure 32.**
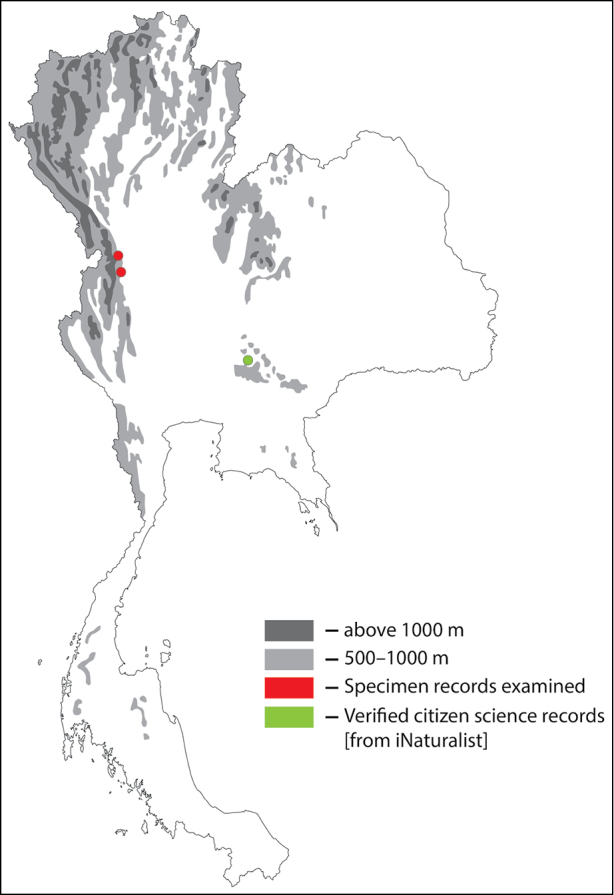
Thai distribution of Megachile (Carinula) stulta.

**Figure 33.**
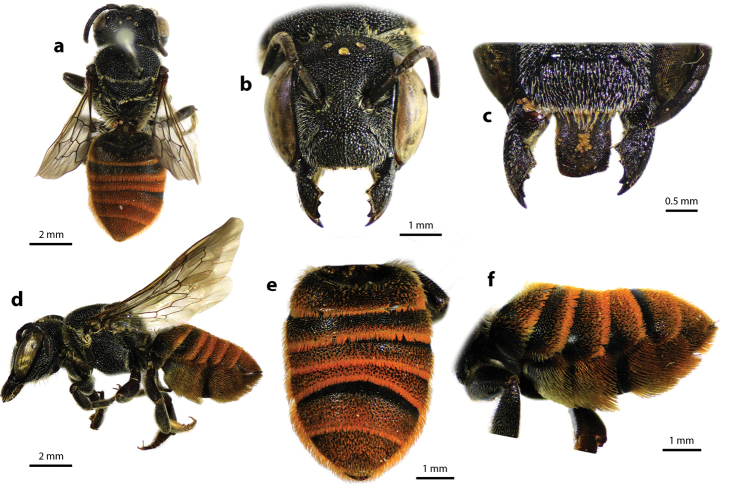
Megachile (Carinula) stulta Bingham, 1897, female **a** dorsal view **b** frontal view **c** frontal view of mandible and labrum **d** lateral view **e** dorsal view of metasoma **f** lateral view of metasoma and scopa.

#### Literature records.

India. Karnataka, Sikkim (Ascher et al. 2016; Ascher and Pickering 2020); Indonesia. Sumatra (Bingham 1897; Ascher and Pickering 2020); Malaysia. Kuala Lumpur, Selangor (Ascher et al. 2016; Ascher and Pickering 2020); Myanmar. Tenasserim (Bingham 1897); Singapore. (Ascher et al. 2016). In addition, six females of this species was among numerous megachilids photographed together at Hin Tung, Mueang District, Nakhon Nayok Province (iNaturalist) (credit: scottyastro 2015; shuanda 2019).

#### Material examined.

Female syntype. India. “Type; B.M. TYPE. 17.a.2161b; *Megachile stulta* Bingh, female, Type.; SIKKIM, Rungjit Valley, 1000 ft., 4.94, BINGHAM COLL., Col. C. T. Bingham 96–30; NHMUK 013380269”; Thailand. Kamphaeng Phet Province: 1♀, Khlong Lan, Khlong Lan waterfall, 08-IV-2014, coll. C. Wimolsuthikul & S. Wongvilas (leg. NC and NW); 36♀, Pang Sila Thong district, Mae Wong National Park, Kang Pha Khoi Nang, 07-VIII-2015, coll. N. Warrit et al. (leg. NC and NW).

##### Notes on *Callomegachile* from Thailand

Two of the most common species of Megachile (Callomegachile) sensu lato found in Thailand are M. (Callomegachile) disjuncta and M. (Callomegachile) umbripennis, and these are also the most common species of this group in Singapore (Ascher et al. 2016). Megachile (Callomegachile) disjuncta is usually collected from *Crotalaria juncea* L. across most of Thailand.

**Figures 34.**
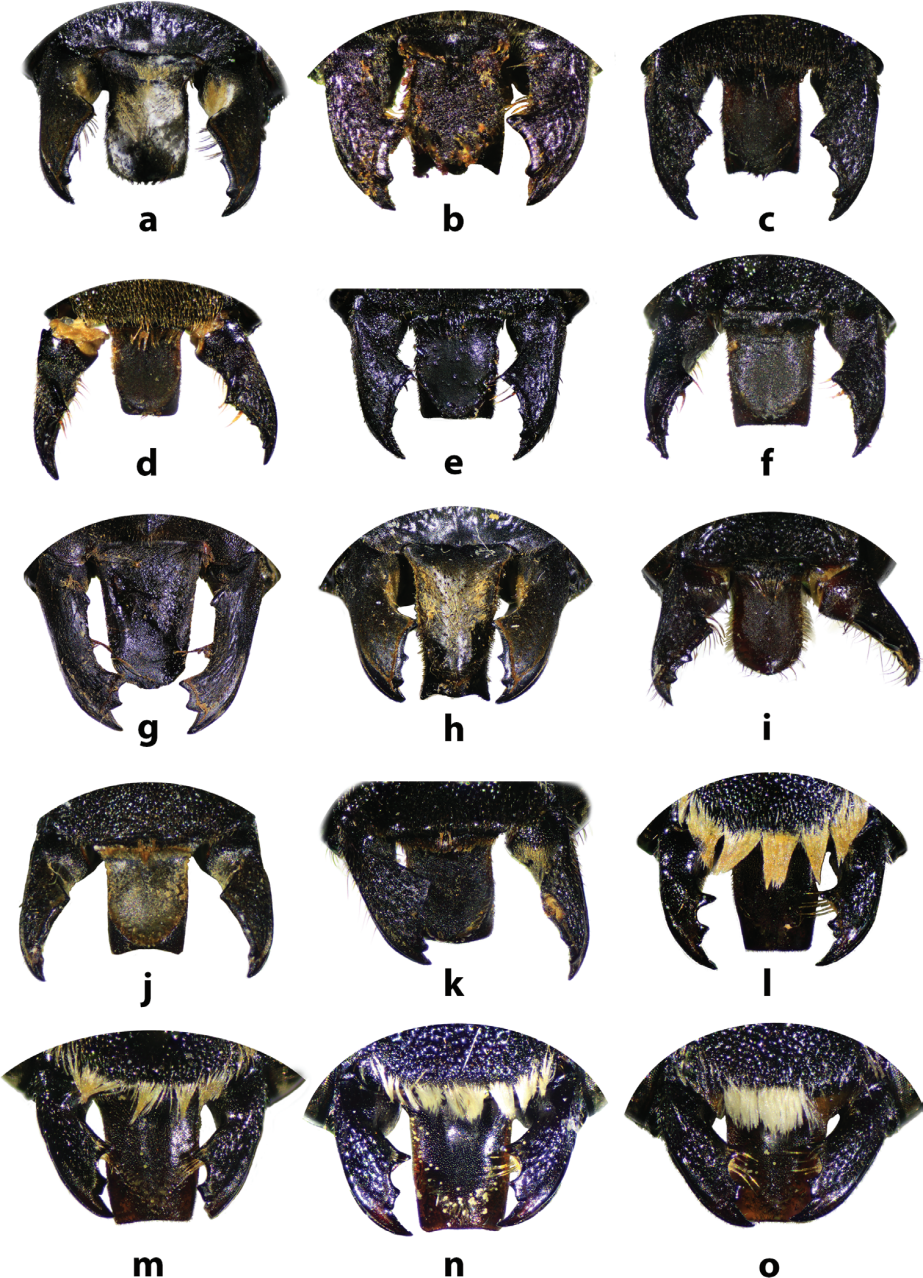
Distal edges of labrums and mandibles of some Thai *Callomegachile* (**a–k** females, **l–o** males) **a**M. (Callomegachile) atratiformis**b**M. (Callomegachile) chiangmaiensis sp. nov. **c, l**M. (Callomegachile) disjuncta**d, m**M. (Callomegachile) faceta**e**M. (Callomegachile) fulvipennis**f**M. (Callomegachile) impressa**g**M. (Callomegachile) monticola**h**M. (Callomegachile) odontophora**i**M. (Callomegachile) parornata**j**, **n**M. (Callomegachile) umbripennis**k**M. (Callomegachile) umbripennis (BSRU AA-4620) **o**M. (Callomegachile) umbripennis (BSRU AA-3654, BSRU AA-3662 and KKIC-02).

This is the first study to emphasize the importance of labral shape (Fig. 34a–o) for the identification in female *Callomegachile* species. In Thailand, M. (Callomegachile) disjuncta is the only species that has two prominent lateral teeth on distal edge of labrum (Fig. 34c), whereas in M. (Callomegachile) fulvipennis, M. (Callomegachile) impressa and M. (Callomegachile) faceta the lateral teeth are less prominent (Fig. 34e, f, d). Megachile (Callomegachile) umbripennis in Thailand has smooth and slightly concave distal edge of labrum (Fig. 34j). Megachile (Callomegachile) parornata has convex distal edge of labrum and M. (Callomegachile) chiangmaiensis sp. nov. has a distinct distal edge: medially convex with large lateral teeth (Fig. 34b). Further investigation into the applicability of the labral distal edge as diagnostic character in other *Callomegachile* species should be carried out.

**Figure 35.**
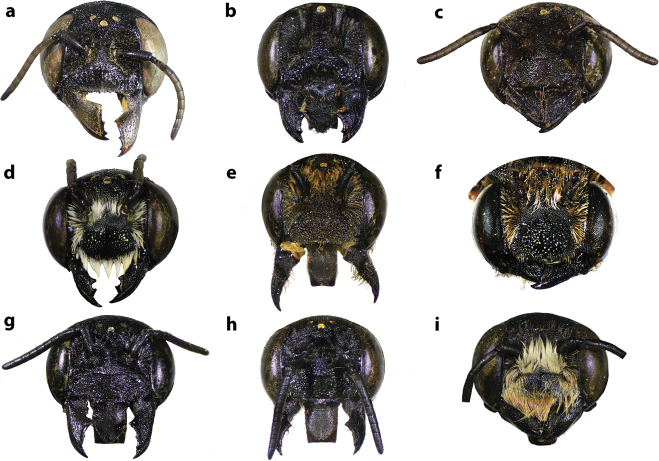
Frontal view of some Thai *Callomegachile* (**a–c, e, g, h** females, **d, f, i** males) **a**M. (Callomegachile) atratiformis**b**M. (Callomegachile) chiangmaiensis sp. nov. **c, d**M. (Callomegachile) disjuncta**e, f**M. (Callomegachile) faceta**g**M. (Callomegachile) fulvipennis**h**M. (Callomegachile) impressa**i**M. (Callomegachile) memecylonae.

**Figure 36.**
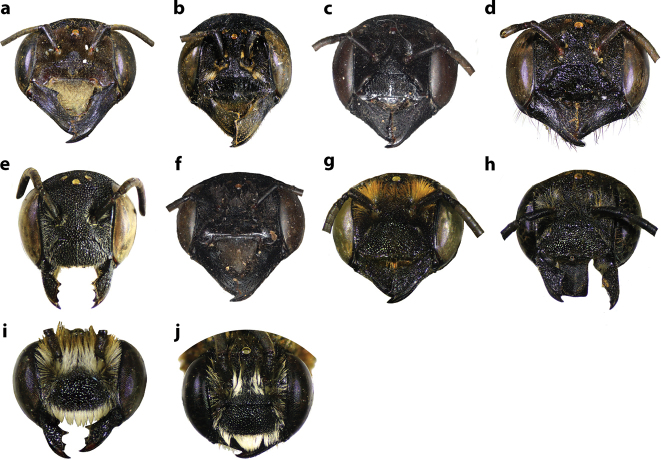
Frontal view of some Thai *Callomegachile* (**a–h** females, **i, j** males) **a**M. (Callomegachile) monticola**b**M. (Callomegachile) odontophora**c**M. (Callomegachile) ornata**d**M. (Callomegachile) parornata**e**M. (Carinula) stulta**f**M. (Callomegachile) tuberculata**g, i**M. (Callomegachile) umbripennis**h**M. (Callomegachile) umbripennis (BSRU AA-4620) **j**M. (Callomegachile) umbripennis (BSRU AA-3654, BSRU AA-3662 and KKIC-02).

**Figure 37.**
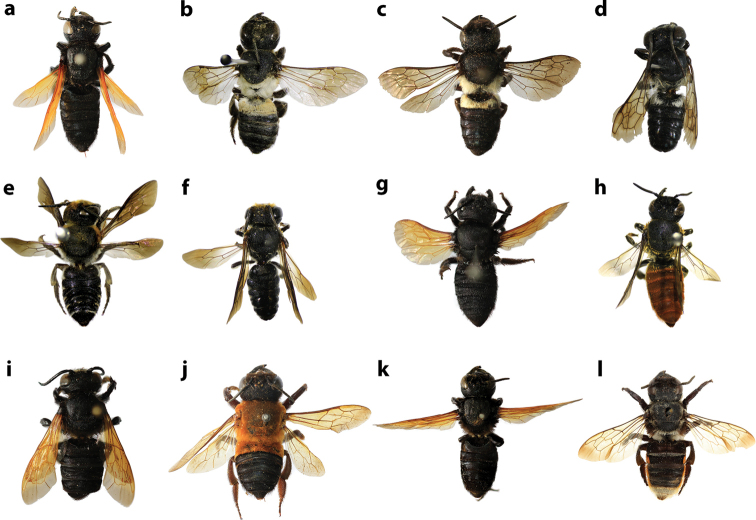
Dorsal view of some Thai *Callomegachile* (**a–c, e, g, h, j–l** females, **d, f, i** males) **a**M. (Callomegachile) atratiformis**b**M. (Callomegachile) chiangmaiensis sp. nov. **c, d**M. (Callomegachile) disjuncta**e, f**M. (Callomegachile) faceta**g**M. (Callomegachile) fulvipennis**h**M. (Callomegachile) impressa**i**M. (Callomegachile) memecylonae**j**M. (Callomegachile) monticola**k**M. (Callomegachile) odontophora**l**M. (Callomegachile) ornata.

**Figure 38.**
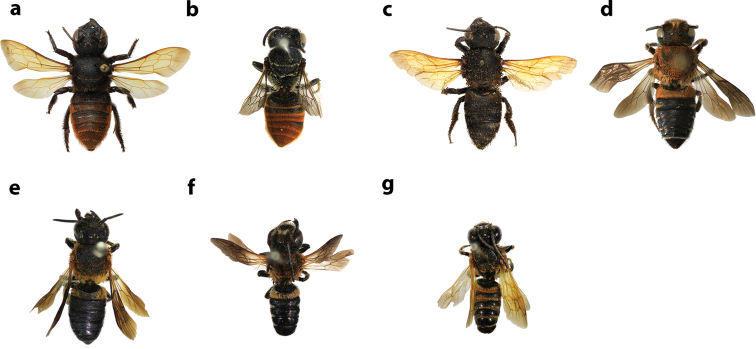
Dorsal view of some Thai *Callomegachile* (**a–e** females, **f, g** male) **a**M. (Callomegachile) parornata**b**M. (Carinula) stulta**c**M. (Callomegachile) tuberculata**d, f**M. (Callomegachile) umbripennis**e**M. (Callomegachile) umbripennis (BSRU AA-4620) **g**M. (Callomegachile) umbripennis (BSRU AA-3654, BSRU AA-3662 and KKIC-02).

### Key to female species of subgenera *Callomegachile* and *Carinula* in Thailand

**Table:** 

1	Pronotum, mesoscutum, and scutellum covered with white and/or black hairs	**2**
–	At least pronotum or mesoscutum and scutellum covered and/or fringed with fulvous hairs	**12**
2	Most metasomal terga covered with red brick hairs or covered with black hairs with fringe of red brick hairs on lateral areas of T2–T5 (Figs 20a, 22d); scopa brick-red	**3**
–	Most metasomal terga covered with black hairs (sometimes T1 with band of white hairs (Figs 2a, 4a); scopa black	**6**
3	Second spine of pro- and mesotibiae bifurcate (Figs 20c, 22f)	**4**
–	Second spine of pro- and mesotibiae not bifurcate	**5**
4	T1–T4 covered with black hairs, T2 with small patch of brick-red hairs laterally; T5–T6 covered with pale light yellow hairs (Fig. 20e)	***M. ornata***
–	T1 covered with black hairs; T2–T5 cover with brick-red hairs; T6 covered with light yellow hairs (Fig. 22g, h)	***M. parornata***
5	Clypeal margin with two small tubercles; mandible five teeth with two stout apical teeth and three small teeth basally	***M. impressa***
–	Clypeal margin crenulate with median carina (Fig. 33b, c); mandible four teeth	***M. stulta***
6	T1 and propodeum covered with white hairs	**7**
–	T1 and propodeum covered with black hairs	**8**
7	Clypeus with prominent apical impression and strong median carina (Fig. 31e); mandible with three stout teeth at apex; labrum oblong, apical margin medially strongly pointed with two lateral teeth (Fig. 31f)	***M. chiangmaiensis* sp. nov.**
–	Clypeus without apical impression, apical margin smooth with two tubercles (Fig. 4d); mandible five teeth with two stout apical teeth at apex and three small teeth basally; labrum rectangle, apical margin truncate with two lateral teeth (Fig. 4e)	***M. disjuncta***
8	Base of clypeus with large protruding tubercle (Fig. 16b); mandible three teeth with small tubercle at base	***M. tuberculata***
–	Base of clypeus without large protruding tubercle; mandible four to five teeth without small tubercle at base	**9**
9	Mandible with five teeth with two stout apical teeth at apex and three small teeth basally; medium size (15–16 mm)	***M. fulvipennis***
–	Mandible with four teeth; large size (20–23 mm)	**10**
10	Apical margin of clypeus with small median tubercle (Fig. 18e); labrum oblong, apical pointed with two lateral teeth (Fig. 18d)	***M. odontophorum***
–	Apical margin of clypeus without median tubercle; labrum rectangle, apical margin truncate without teeth	**11**
11	Mesoscutum with strong transverse wrinkle pattern on disc, posteriorly with irregular punctures	***M. memecylonae***
–	Mesoscutum with weak transverse wrinkle pattern on disc, posteriorly with weakly transverse wrinkle pattern-like disc	***M. atratiformis***
12	Fulvous or black tergal hair bands; base of clypeus with large protruding tubercle (Fig. 16b); mandible three teeth with small tubercles at base (Fig. 16c); scopa black; large size (20–26 mm)	***M. monticola***
–	White tergal hair bands, sometimes interrupted at median, base of clypeus without large protruding tubercle; mandible five teeth with two stout apical teeth at apex and three small teeth basally without tubercle at base; scopa white with black at apex; median size (10–13 mm)	**13**
13	Vertex with median carina (Fig. 7e); only pronotum covered with fulvous hairs; propodeal triangle with tuft white hairs	***M. faceta***
–	Vertex without median carina; pronotum, mesoscutum, and scutellum covered with dense fulvous hairs throughout; propodeal triangle with dense fulvous hairs	***M. umbripennis***

## Supplementary Material

XML Treatment for Megachile

XML Treatment for Subgenus Callomegachile

XML Treatment for Megachile (Callomegachile) atratiformis

XML Treatment for Megachile (Callomegachile) disjuncta

XML Treatment for Megachile (Callomegachile) faceta

XML Treatment for Megachile (Callomegachile) fulvipennis

XML Treatment for Megachile (Callomegachile) impressa

XML Treatment for Megachile (Callomegachile) memecylonae

XML Treatment for Megachile (Callomegachile) monticola

XML Treatment for Megachile (Callomegachile) odontophora

XML Treatment for Megachile (Callomegachile) ornata

XML Treatment for Megachile (Callomegachile) parornata

XML Treatment for Megachile (Callomegachile) tuberculata

XML Treatment for Megachile (Callomegachile) umbripennis

XML Treatment for Megachile (Callomegachile) chiangmaiensis

XML Treatment for Megachile (Carinula)

XML Treatment for Megachile (Carinula) stulta
